# *In silico* modeling predicts drug sensitivity of patient-derived cancer cells

**DOI:** 10.1186/1479-5876-12-128

**Published:** 2014-05-21

**Authors:** Sandeep C Pingle, Zeba Sultana, Sandra Pastorino, Pengfei Jiang, Rajesh Mukthavaram, Ying Chao, Ila Sri Bharati, Natsuko Nomura, Milan Makale, Taher Abbasi, Shweta Kapoor, Ansu Kumar, Shahabuddin Usmani, Ashish Agrawal, Shireen Vali, Santosh Kesari

**Affiliations:** 1 Translational Neuro-Oncology Laboratories, Moores Cancer Center, UC San Diego, La Jolla, CA 92093, USA; 2 Cellworks Research India Ltd, Bangalore 560 066, India; 3 Cellworks Group Inc, Saratoga, CA 95070, USA; 4 Department of Neurosciences, UC San Diego, La Jolla, CA 92093, USA

**Keywords:** Glioblastoma, Cancer, *In Silico* modeling, Deterministic model, Virtual tumor technology, Tumor profiling, Personalized therapy, Targeted therapy

## Abstract

**Background:**

Glioblastoma (GBM) is an aggressive disease associated with poor survival. It is essential to account for the complexity of GBM biology to improve diagnostic and therapeutic strategies. This complexity is best represented by the increasing amounts of profiling (“omics”) data available due to advances in biotechnology. The challenge of integrating these vast genomic and proteomic data can be addressed by a comprehensive systems modeling approach.

**Methods:**

Here, we present an *in silico* model, where we simulate GBM tumor cells using genomic profiling data. We use this *in silico* tumor model to predict responses of cancer cells to targeted drugs. Initially, we probed the results from a recent hypothesis-independent, empirical study by Garnett and co-workers that analyzed the sensitivity of hundreds of profiled cancer cell lines to 130 different anticancer agents. We then used the tumor model to predict sensitivity of patient-derived GBM cell lines to different targeted therapeutic agents.

**Results:**

Among the drug-mutation associations reported in the Garnett study, our *in silico* model accurately predicted ~85% of the associations. While testing the model in a prospective manner using simulations of patient-derived GBM cell lines, we compared our simulation predictions with experimental data using the same cells *in vitro*. This analysis yielded a ~75% agreement of *in silico* drug sensitivity with *in vitro* experimental findings.

**Conclusions:**

These results demonstrate a strong predictability of our simulation approach using the *in silico* tumor model presented here. Our ultimate goal is to use this model to stratify patients for clinical trials. By accurately predicting responses of cancer cells to targeted agents *a priori*, this *in silico* tumor model provides an innovative approach to personalizing therapy and promises to improve clinical management of cancer.

## Introduction

Cancer remains a major unmet clinical need despite advances in clinical medicine and cancer biology. Glioblastoma (GBM) is the most common type of primary adult brain cancer, characterized by infiltrative cellular proliferation, angiogenesis, resistance to apoptosis, and widespread genomic aberrations. GBM patients have poor prognosis, with a median survival of 15 months [1]. Molecular profiling and genome-wide analyses have revealed the remarkable genomic heterogeneity of GBM [2,3]. Based on tumor profiles, GBM has been classified into four distinct molecular subtypes [4]. However, even with existing molecular classifications, the high intertumoral heterogeneity of GBM makes it difficult to predict drug responses *a priori*. This is even more evident when trying to predict cellular responses to multiple signals following combination therapy. Our rationale is that a systems-driven computational approach will help decipher pathways and networks involved in treatment responsiveness and resistance.

Though computational models are frequently used in biology to examine cellular phenomena, they are not common in cancers, particularly brain cancers [5,6]. However, models have previously been used to estimate tumor infiltration following surgery [7] or changes in tumor density following chemotherapy in brain cancers [8]. More recently, brain tumor models have been used to determine the effects of conventional therapies including chemotherapy and radiation [5]. Brain tumors have also been studied using an agent-based modeling approach [9]. Multiscale models that integrate hierarchies in different scales are being developed for application in clinical settings [10]. Unfortunately, none of these models have been successfully translated into the clinic so far. It is clear that innovative models are required to translate data involving biological networks and genomics/proteomics into optimal therapeutic regimens. To this end, we present a deterministic *in silico* tumor model that can accurately predict sensitivity of patient-derived tumor cells to various targeted agents.

## Methods

### Description of *In Silico* model (Version 7.3 Cellworks)

We performed simulation experiments and analyses using the predictive tumor model – a comprehensive and dynamic representation of signaling and metabolic pathways in the context of cancer physiology. This *in silico* model includes representation of important signaling pathways implicated in cancer such as growth factors such as EGFR, PDGFR, FGFR, c-MET, VEGFR and IGF-1R; cytokine and chemokines such as IL1, IL4, IL6, IL12, TNF; GPCR mediated signaling pathways; mTOR signaling; cell cycle regulations, tumor metabolism, oxidative and ER stress, representation of autophagy and proteosomal degradation, DNA damage repair, p53 signaling and apoptotic cascade. The current version of this model includes more than 4,700 intracellular biological entities and ~6,500 reactions representing their interactions, regulated by ~25,000 kinetic parameters. This comprises a comprehensive and extensive coverage of the kinome, transcriptome, proteome and metabolome. Currently, we have 142 kinases and 102 transcription factors modeled in the system.

### Model development

We built the basic model by manually curating data from the literature and aggregating functional relationships between proteins. The detailed procedure for model development is explained in Additional file 1 (Section 2) using the example of the epidermal growth factor receptor (EGFR) pathway block (Additional file 1: Figure S1 and Figure S2). We have also presented examples of how the kinetic parameters are derived from experimental data, in Additional file 1: (Section 2). We have validated the simulation model prospectively and retrospectively, at phenotype and biomarker levels using extensive *in vitro* and *in vivo* studies [11-20].

### Disease phenotype definitions

Disease phenotype indices are defined in the tumor model as functions of biomarkers involved. Proliferation Index is an average function of the active CDK-Cyclin complexes that define cell cycle check-points and are critical for regulating overall tumor proliferation potential. The biomarkers included in calculating this index are: CDK4-CCND1, CDK2-CCNE, CDK2-CCNA and CDK1-CCNB1. These biomarkers are weighted and their permutations provide an index definition that gives maximum correlation with experimentally reported trend for cellular proliferation (based on literature).

We also generate a Viability Index based on 2 sub-indices: Survival Index and Apoptosis Index. The biomarkers constituting the Survival Index include: AKT1, BCL2, MCL1, BIRC5, BIRC2 and XIAP. These biomarkers support tumor survival. The Apoptosis Index comprises: BAX, CASP3, NOXA and CASP8. The overall Viability Index of a cell is calculated as a ratio of Survival Index/Apoptosis Index. The weightage of each biomarker is adjusted so as to achieve a maximum correlation with the experimental trends for the endpoints (based on literature).

In order to correlate the results from experiments such as MTT Assay, which are a measure of metabolically active cells, we have a ‘Relative Growth’ Index that is an average of the Survival and Proliferation Indices.

The percent change seen in these indices following a therapeutic intervention helps assess the impact of that particular therapy on the tumor cell. A cell line in which the Proliferation/Viability Index decreases by <20% from the baseline is considered resistant to that particular therapy.

### Creation of cancer cell line and its variants

To create a cancer-specific simulation model, we start with a representative non-transformed epithelial cell as control. This cell is triggered to transition into a neoplastic state, with genetic perturbations like mutation and copy number variation (CNV) known for that specific cancer model. We also created *in silico* variants for cancer cell lines, to test the effect of various mutations on drug responsiveness. We created these variants by adding or removing specific mutations from the cell line definition. For example, DU145 prostate cancer cells normally have RB1 deletion. To generate a variant of DU145 with wild-type RB1 (WT), we retained the rest of its mutation definition except for the RB1 deletion, which was converted to WT RB1 (Additional file 1).

### Simulation of drug effect

To simulate the effect of a drug in the *in silico* tumor model, the targets and mechanisms of action of the drug are determined from published literature. The drug concentration is assumed to be post-ADME (Absorption, Distribution, Metabolism and Excretion).

### Creation of simulation avatars of patient-derived GBM cell lines

**Figure 1:**
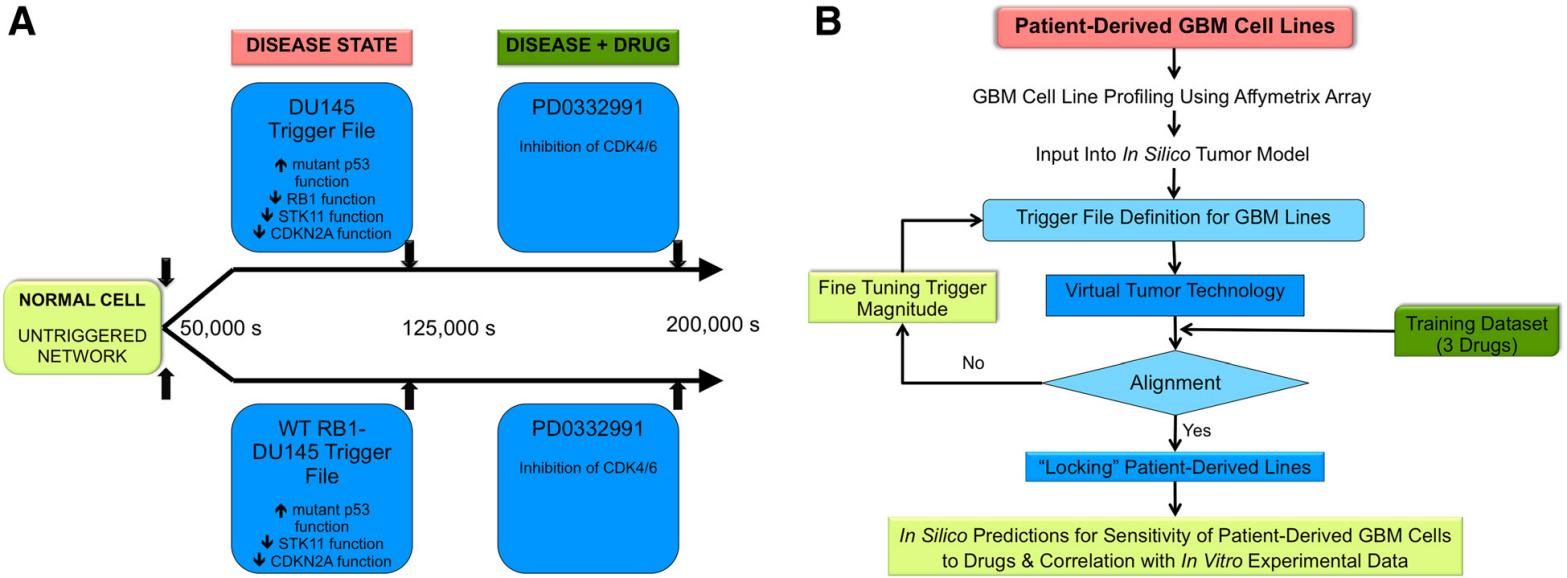
**Simulation workflow for*in silico*tumor model. A**, This illustration depicts a representative simulation protocol used for retrospective analysis of gene mutation-drug sensitivity association reported in the Garnett study. The simulation protocol included 3 states: Control or Untriggered state is simulated for 50,000 seconds to allow the biological entities to attain a steady-state concentration. At 50,000 seconds, mutation data is introduced and simulated for an additional 1,25,000 seconds to attain Disease state. For Drug-treated state, we introduce a drug into the system by perturbing the target reaction nodes and simulate the model for 2,00,000 seconds. At the end of this state, we calculate percent change in the indices for cell survival. **B**, This schematic demonstrates the simulation workflow for creation, optimization and testing of patient-derived GBM cell line profiles *in silico*. The key steps involved in developing the simulation avatars of the patient-derived GBM cell lines include: Input profiling data reporting relative expression of the different proteins in the cell lines; Iterative testing and alignment of simulation avatars to match experimental data on drugs used for alignment of the network (erlotinib, sorafenib and dasatinib); Locking the simulation avatars; *In silico* predictions and *in vitro* testing.

To predict drug sensitivity in patient-derived GBM cell lines, we created simulation avatars (*in silico* profiles) for each cell line as illustrated in Figure 1B. First, we simulated the network dynamics of GBM cells by using experimentally determined expression data (Additional file 1: Table S1; Additional file 1: Section 7). Next, we overlay tumor-specific genetic perturbations on the control network, in order to dynamically generate the simulation avatar. For instance, the patient-derived cell line SK987 is characterized by overexpression of AKT1, EGFR, IL6, and PI3K among other proteins and knockdown of CDKN2A, CDKN2B, RUNX3, etc. (Additional file 1: Table S1). After adding this information to the model, we further optimized the magnitude of the genetic perturbations, based on the responses of this simulation avatar to three molecularly targeted agents: erlotinib, sorafenib and dasatinib. The response of the cells to these drugs (from *in vitro* experimental data) was used as an “alignment data set”. In this manner, we used “alignment drugs” (erlotinib, sorafenib, and dasatinib) to optimize the magnitude of genetic perturbation in the trigger files and their impact on key pathways targeted by these drugs. For example, most GBM cell lines demonstrated dominance of EGFR signaling as they had gains in copy number of EGFR gene. Hence the effect of EGFR inhibitor would be a good indicator for the relative dominance of this signaling pathway. This is illustrated in further details in Additional file 1 using an example of two cell line profiles that have EGFR over-expression but differential response to EGFR inhibitor. Similarly, sorafenib helped determine and align with MEK/ERK activation, while dasatinib with activation of SRC signaling.

### Simulation protocol

The simulation protocol included 3 states:

1. Control State – The *in silico* model was simulated for 50,000 seconds, during which the different biological entities (called species) attain a steady-state concentration. This concentration depends on the balance between the rate of reaction nodes *producing the species* and the reaction nodes *utilizing/degrading the species*. This is an untriggered system and is representative of a non-transformed epithelial cell.

2. Disease State – At 50,000 seconds simulation time, we introduced the mutation data (specific to patient-derived GBM cell lines to be created) and simulated for an additional 1,25,000 seconds. During this time, the system attained a new steady state that aligns to the network dynamics of the cell line.

3. Disease State + Drug Treatment – Following the simulation run time of 1,25,000 seconds for Disease State, we introduced the drug into the system by perturbing the target reaction nodes as explained above. We then simulated the model further for 2,00,000 seconds (drug treatment). A percent change in the indices for cell survival (described earlier) indicates the therapeutic potential of the drug. Iterative simulations with varying concentrations of the drug generate dose-response curves from which IC_50_ values can be determined.

Figure 1A is a schematic of the representative simulation protocol that we used for the retrospective analysis of gene mutations-drug effects reported in the study by Garnett and co-workers. Figure 1B illustrates the workflow for simulation studies on patient-derived GBM cell lines. For the patient-derived GBM cell line predictions, we prospectively compared *in silico* responses to experimentally obtained results (*in vitro* data from patient-derived GBM cell lines) and determined corroboration between *in silico* and *in vitro* data. As per the dose-response plots generated by *in silico* predictions, a cell line was considered sensitive to a drug if it demonstrated >20% decrease in relative growth. The 20% threshold was used for both *in silico* predictions and for *in vitro* experimental data.

### Patient-derived glioblastoma cell lines

Fresh human glioblastoma samples were acquired from brain tumor patients undergoing clinically indicated surgery (University of California San Diego Human Subjects Protocol) and cultured as previously reported [21,22]. GBM4 and 8 cells were a kind gift from C. David James (University of California San Francisco). Briefly, the dissociated tissue was washed, filtered through a 30 μm mesh and plated onto ultra-low adherence flasks at a concentration of 500,000 to 1,500,000 viable cells/ml. The stem cell isolation medium included human recombinant EGF (20 ng/ml), human bFGF (10 ng/ml) and heparin (2 μg/ml). Sphere cultures were passaged by dissociation using Acutase (Sigma), washed, resuspended in neural stem cell culture medium (#05750, Stemcell Technologies), and plated on ultra low-adherence 96 well plates at 2000 cells per well for all subsequent drug testing. We characterized all patient-derived glioblastoma lines using histopathologic and integrated genomic analyses. The glioblastoma lines were profiled using the Affymetrix Gene Chip Human Gene 1.0 ST Array.

### Drug screening

Drug screens were performed on patient-derived GBM cell lines plated at 2000 cell per well in 96-well microtiter plates, incubated overnight. After 72 hours of incubation with drugs, cell viability was quantified by the Alamar Blue assay. Briefly, after incubation, Alamar Blue (#BUF012B, AbDSerotec) was added directly to the culture medium, and the fluorescence measured at 560/90 to determine the number of viable cells (Infinite M200, Tecan Group Ltd.).

## Results

Our study involved a retrospective component where we predicted gene mutations – drug sensitivity associations defined in a recent hypothesis-independent study [23]. In addition, we predicted sensitivity of our profiled patient-derived GBM cell lines to targeted agents and compared these *in silico* predictions to *in vitro* experimental data.

### Retrospective validation of *in Silico* tumor model

In the first part of the study, we evaluated the ability of the *in silico* tumor model to predict drug responses that were reported in the study by Garnett and colleagues [23]. A comparison of our predictions with the associations reported in the Garnett study indicated the predictive capability of our *in silico* tumor model.

Our modeling library has definitions for 45 of the 639 cell lines used in this study (Additional file 1: Table S2) and supports 70 of the 130 drugs studied (Additional file 1: Table S3). Further, we can represent 51 of the 84 genes screened for mutations (Additional file 1: Table S4). Of the 448 significant gene mutation-drug response associations reported, our *in silico* model was able to accurately predict 22 of the 25 testable associations from the Garnett study (>85% agreement; Additional file 1: Table S5). The gene mutation–drug response correlations from the Garnett study that are currently not supported by the system are listed in Additional file 1: Table S6. From the 25 gene mutation–drug response associations tested from the Garnett study (Additional file 1: Table S5), a few examples of the correlations are explained below. Figure 1A depicts a representative schematic of this retrospective analysis using the simulation (*in silico* tumor model).

### BRAF Mutations and Drug Sensitivity

The Garnett study showed that cells with BRAF mutation were sensitive to the MEK1/2 inhibitor AZD2644 [23]. To examine this association, we modeled cancer cell variants with wild-type BRAF *in silico*. Modeling data showed that cells with wild-type BRAF were resistant to AZD6244, when compared to the parent tumor cells with mutant BRAF. Thus, BRAF mutation conferred sensitivity to the MEK1/2 inhibitor *in silico*; this prediction validates the finding reported in the Garnett study (Figure 1A). 40-60% melanoma patients carry BRAF mutations that activate MAPK signaling [24,25] and this association could have therapeutic implications for the treatment of patients with BRAF mutant melanoma.

### Effect of different mutations on sensitivity to tyrosine Kinase inhibitors

**Figure 2:**
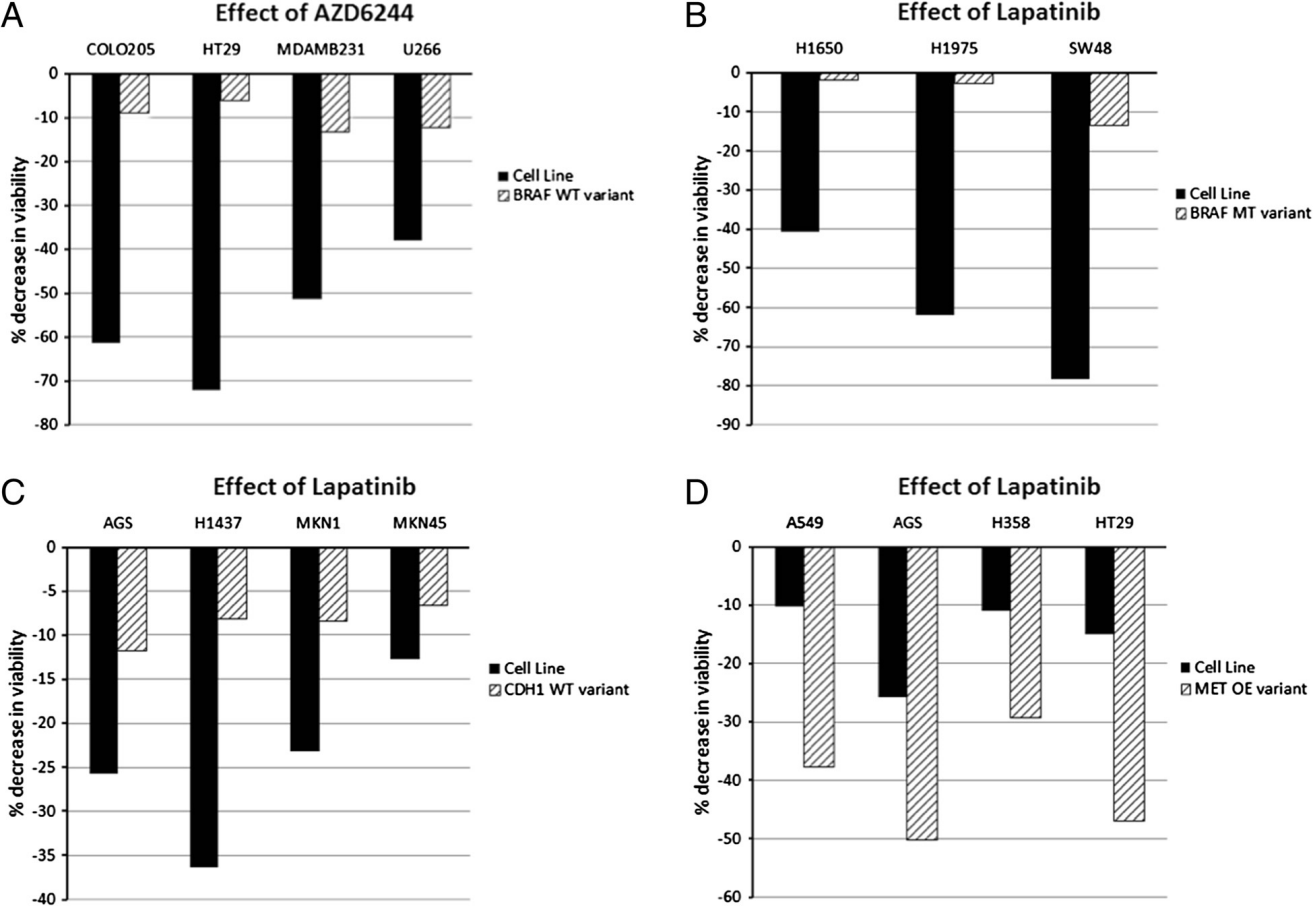
**Retrospective analysis tests*in silico*predictions of gene mutations and sensitivity to EGFR family inhibitors.** Associations reported in the Garnett study were tested in a blinded manner using our *in silico* model and predictions obtained were compared to results reported in the Garnett study. **A**, We created wild-type BRAF variants of four cancer cell lines – COLO205, HT29, MDAMB231 and U266 *in silico* and compared the effect of MEK1/2 inhibitor AZD2644 on these cell lines and on corresponding parent lines expressing mutant BRAF. Our data demonstrated that BRAF mutation increases sensitivity to AZD6244. **B**, We simulated three cell lines – H1650, H1975 and SW48 with wild-type or mutant BRAF and tested for sensitivity to the EGFR2 family kinase inhibitor, lapatinib. BRAF mutation decreases sensitivity of cells to lapatinib. **C**, Similarly, when four cell lines (AGS, H1437, MKN1 and MKN45) were tested for sensitivity to lapatinib, we observed that CDH1 mutation increases sensitivity to lapatinib. **D**, We generated cell lines with wild-type or MET over-expression and tested the effect of lapatinib (A549, AGS, H358 and HT29 cell lines). MET over-expression increases sensitivity to lapatinib.

The Garnett study showed that cells with BRAF mutation were sensitive to the MEK1/2 inhibitor AZD2644 [23]. To examine this association, we created cancer cell variants with wild-type BRAF in the *in silico* model. Simulation data showed that cells with wild-type BRAF were resistant to AZD6244, when compared to cells with mutant BRAF. Thus, BRAF mutation conferred sensitivity to the MEK1/2 inhibitor; this validates the finding reported in the Garnett study (Figure 2A). 40-60% melanoma patients carry BRAF mutations that activate MAPK signaling [24,25]. This association tested in Figure 2A may have therapeutic implications for the treatment of patients with BRAF mutant melanoma.

ERBB2 (HER2) amplification is a biomarker for sensitivity to EGFR-family inhibitors [26]. In the *in silico* model, we tested for sensitivity to EGFR2 family inhibitors, lapatinib and BIBW2992. Specifically, we examined sensitivity of cancer cells in the presence of mutations and/or over-expression of BRAF, CDH1, ERBB2, CCND1 and MET. These predictions from simulations were compared with results obtained in the Garnett study and the predictive capability of our model was determined.

*In silico* predictions indicate that BRAF mutation decreases sensitivity of cells to lapatinib (Figure 2B), whereas CDH1 mutant lines demonstrated higher sensitivity to lapatinib when compared to variants with wild-type CDH1 (Figure 2C). Further, cMET over-expression showed increased sensitivity to lapatinib, as indicated by decrease in viability in cells with cMET over-expression (Figure 2D). Additionally, ERBB2 and CCND1 over-expression correlated positively with lapatinib sensitivity (Additional file 1: Table S5). In all these simulation experiments testing sensitivity to lapatinib, our *in silico* predictions corroborated with associations reported in the Garnett study.

### CDKN2A mutation and drug sensitivity

**Figure 3:**
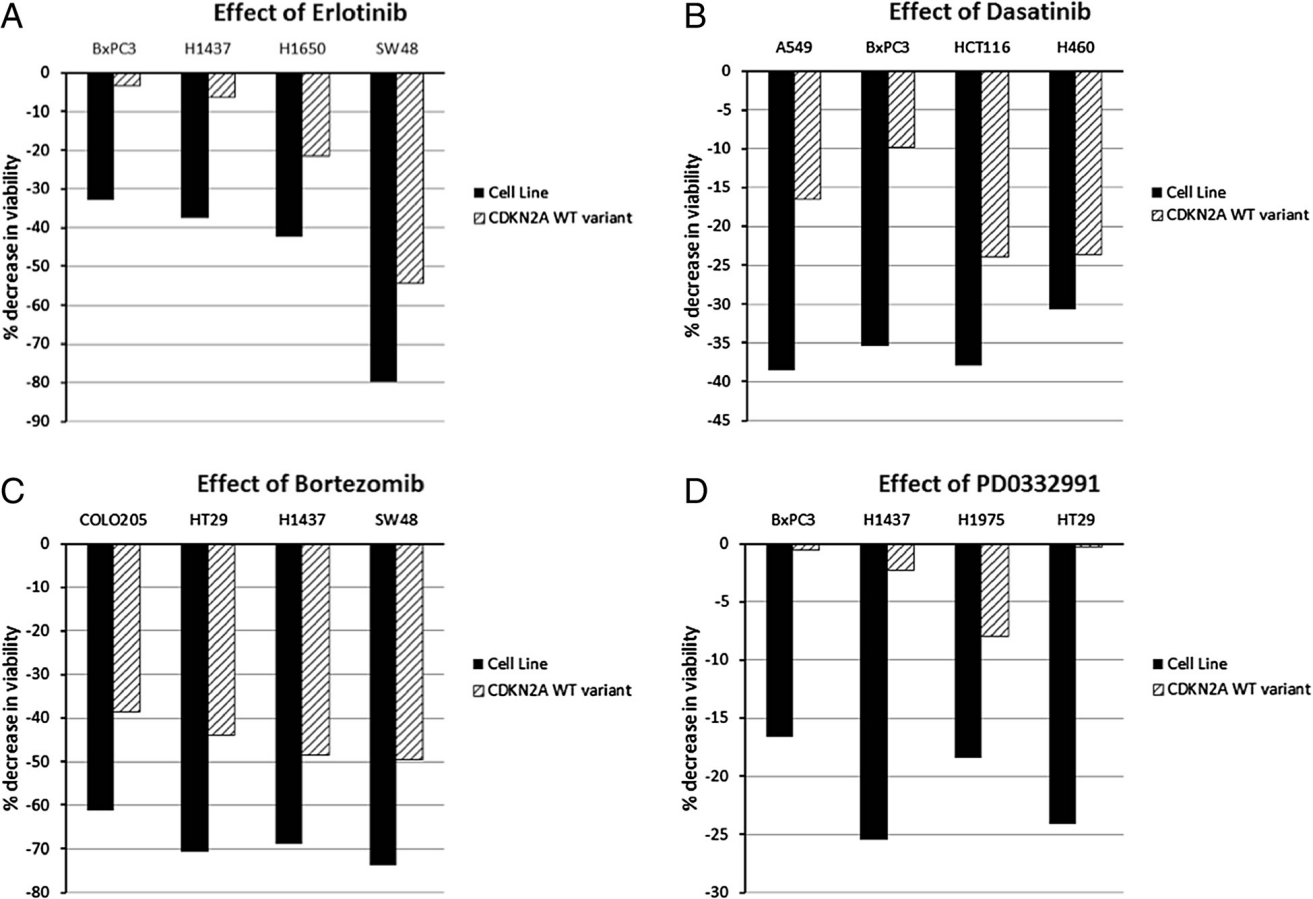
**Retrospective analysis evaluates CDKN2A mutation – drug response association by*in silico*modeling.** Using simulation modeling, we tested the role of the tumor-suppressor protein CDKN2A on sensitivity to different inhibitors and compared these predictions to those reported in the Garnett study. **A**, Cells expressing mutant CDKN2A and their wild-type variants were simulated in the *in silico* tumor model for four lines – BxPC3, H1437, H1650 and SW48. CDKN2A mutation increased sensitivity of cells to erlotinib when compared to wild-type CDKN2A. **B**, Cells with mutant CDKN2A were more sensitive to dasatinib than cells with wild-type CDKN2A (A549, BxPC3, HCT116 and H460). **C**, COLO205, HT29, H1437 and SW48 cell lines with mutant CDKN2A were sensitive to bortezomib more than cells expressing wild-type variants. **D**, CDKN2A mutant cells BxPC3, H1437, H1975 and HT29 also showed higher sensitivity to CDK4-Cyclin D1 inhibitor PD0332991 over the CDKN2A WT variants.

The Garnett study reported associations between tumor suppressor gene mutations and several anti-cancer drugs. We tested these associations in our *in silico* tumor model. In the *in silico* analysis, cells harboring wild-type CDKN2A were resistant to erlotinib whereas CDKN2A mutation was associated with erlotinib sensitivity (Figure 3A). Similarly, cell lines with mutant CDKN2A showed increased sensitivity to dasatinib (Figure 3B), bortezomib (Figure 3C), and to the CDK4/6 inhibitor PD0332991 (Figure 3D). These predictions/analyses from our simulation corroborated accurately with data from the Garnett study.

Other gene mutation-drug response associations examined in our simulation models are illustrated in Additional file 1: Table S5. In addition, Additional file 1: Table S6 lists correlations between gene mutations and drug responses reported in the Garnett study, which are currently not supported by our modeling technology. In spite of these limitations, we obtained ~85% agreement of our simulation data with findings reported by Garnett [23].

### Prospective evaluation of tumor model – patient-derived GBM cell lines

Identifying drug sensitivities in tumors/cancers with different mutations is important for designing individualized therapies for cancer. To this end, we created *in silico* avatars of 8 patient-derived GBM cell lines using genomic data (Methods and Additional file 1: Table S1) and predicted their sensitivity to various targeted therapeutic agents. We then tested these *in silico* predictions prospectively by comparing then with experimental data obtained by *in vitro* testing on the same patient-derived GBM cell lines (Figure 1B).

The patient-derived GBM cell lines were obtained from patient tumors resected surgically and cultured *in vitro* (details in Methods). We have profiled these lines using Affymetrix Gene Chip Human Gene 1.0 ST Array. Using whole-exome sequencing, we recently tested the validity of these cells (maintained in cultures) for development and testing of personalized targeted therapies, based on their accurate representation of the original tumor profiles [27]. We have designated the different patient-derived GBM cell lines as: GBM4, GBM8, SK102, SK262, SK429, SK748, SK987 and SK1035.

**Figure 4:**
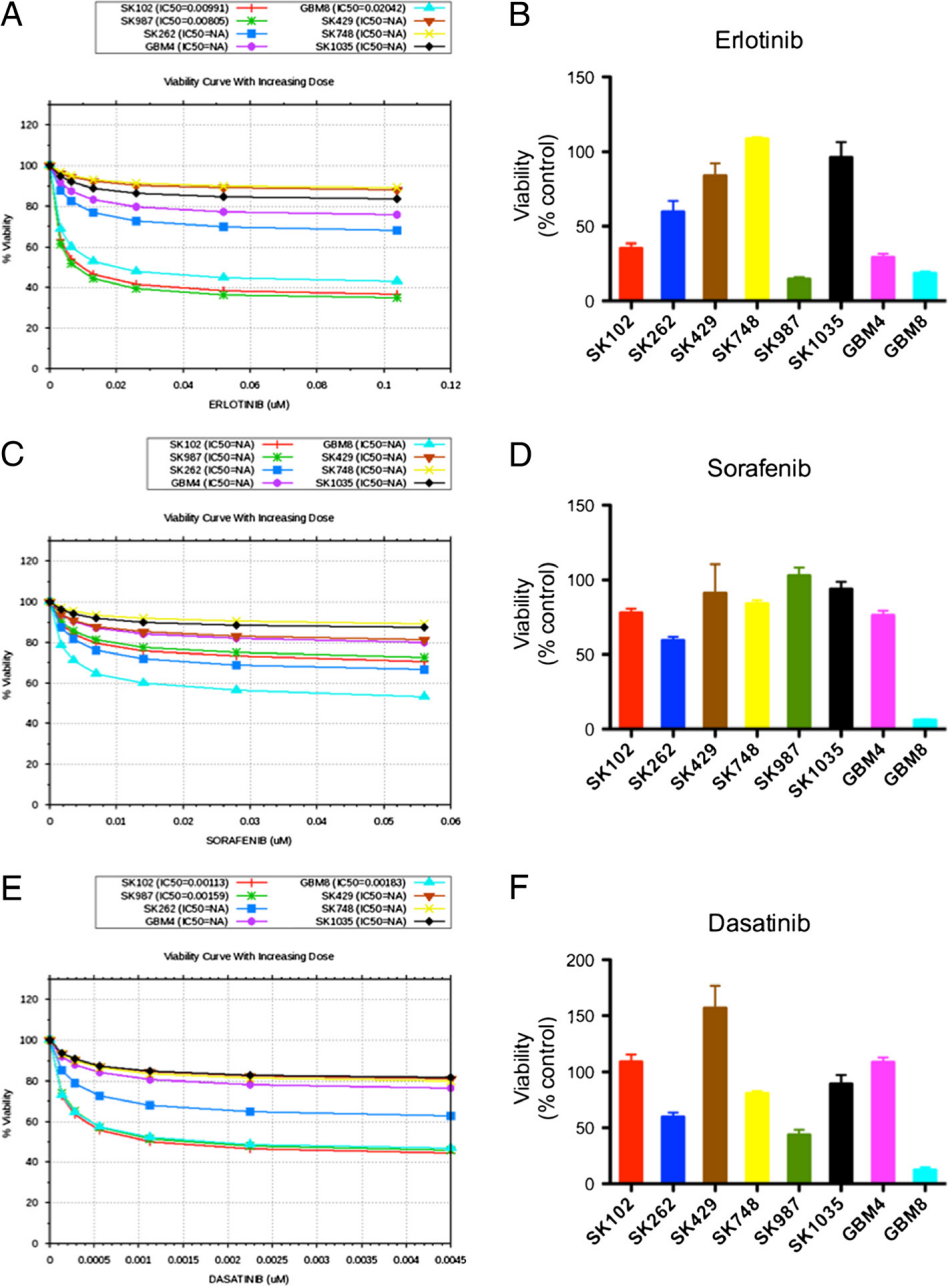
***In silico*modeling analysis and experimental*in vitro*data for drug responsiveness to 3 alignment drugs. A**, Predictive dose response data for erlotinib with percent change in viability. Cells showing decrease in viability of 20% or greater are considered sensitive to the drug. **B**, *In vitro* experimental results for effect of 1 μM erlotinib on viability in patient-derived GBM cell lines; viability was determined at 72 h using Alamar Blue assay. **C**, **D**, Predictive and experimental data for sorafenib. **E**, **F**, Predictive and experimental data for dasatinib. All drugs were tested *in vitro* at 1 μM. Dose-response curves for *in silico* data demonstrate the effects of increasing concentrations of the drugs – erlotinib, sorafenib and sunitinib on the viability of profiled patient-derived GBM cell lines in the simulation model.

After generating *in silico* profiles of these cells, we optimized these simulation avatars in terms of strength of functional effect of the mutation on key pathways such as EGFR, RAS and Src/PI3K. The rationale for this optimization is that expression data on these cells does not provide an accurate measure of the dominance of different intracellular pathways. In order to interrogate this information on the pathways that play a dominant role in each tumor line (such as EGFR, RAS, PI3K, etc.), we used 3 anti-cancer agents (erlotinib, sorafenib and dasatinib) targeting these pathways. This will achieve “alignment” and train the simulation avatars for further analyses (details in Additional file 1). The alignment for these 3 drugs could be best achieved in the following cell lines: GBM8, SK262, SK429, SK748, and SK1035. In cell lines GBM4 and SK987, there was a mismatch for sorafenib where the predictive trends were reversed. GBM4 was sensitive to sorafenib experimentally but our *in silico* predictions showed it to be resistant; SK987 was resistant experimentally but sensitive in predictive results. Similarly, the experimental trend for SK102 resistance to dasatinib could not be met predictively. Correlation of predictive trends with alignment drugs is shown in Figure 4 A-F.

Predictions obtained by simulation modeling are presented as dose-response plots for viability; decrease in viability of >20% was considered as sensitive. Experimentally, viability was determined by Alamar blue assay, in response to 1 μM concentration of respective inhibitors at 72 h. These data represent viability as mean values from triplicate samples.

**Figure 5:**
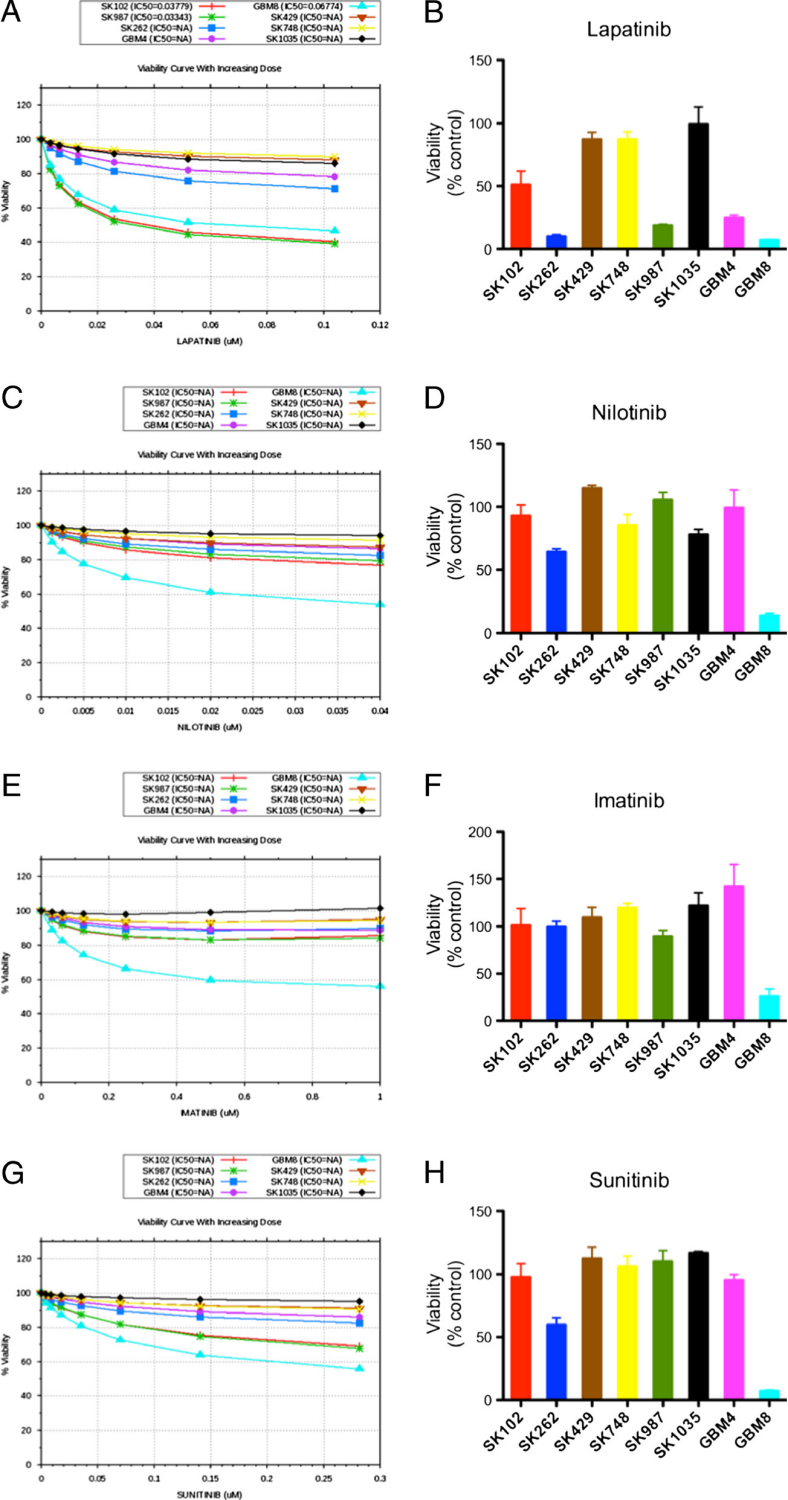
***In silico*modeling and experimental*in vitro*data for drug responsiveness to tyrosine kinase inhibitors.** This figure demonstrates *in silico* predictions of sensitivity and *in vitro* viability (respectively) in response to treatment with tyrosine kinase inhibitors: **A**, **B**, lapatinib, **C**, **D**, nilotinib, **E**, **F**, Imatinib and **G**, **H**, Sunitinib. Cells were exposed *in vitro* to 1 μM tyrosine kinase inhibitors for 72 h and viability determined using Alamar Blue assay. The dose-response for *in silico* predictions is generated by iterative simulations with increasing concentrations of the drug in the model and the viability index is calculated. Cells showing decrease in viability of 20% or greater are considered sensitive to the drug.

**Figure 6:**
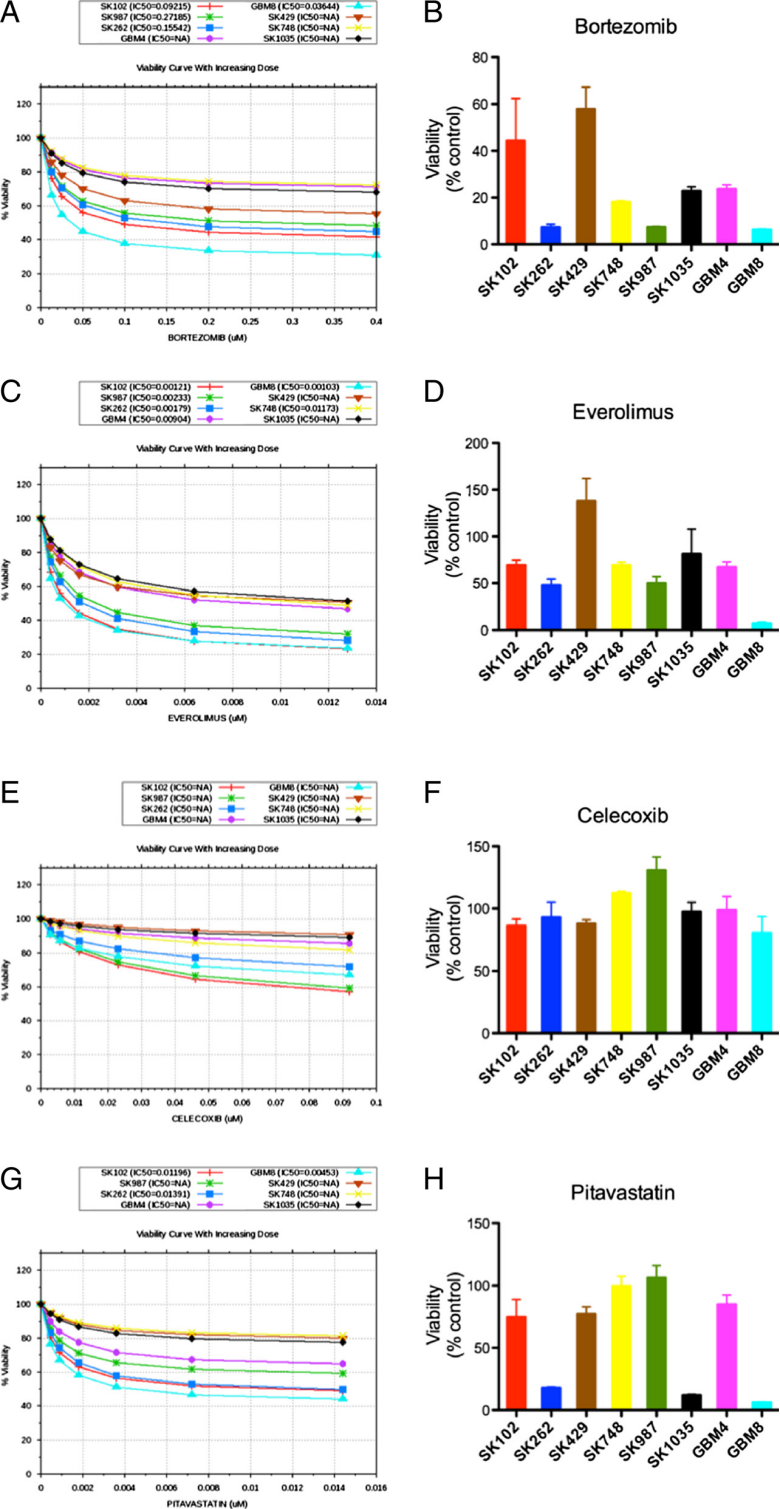
***In silico*modeling and experimental*in vitro*data for drug responsiveness to different drugs.** This figure demonstrates *in silico* predictions of sensitivity and *in vitro* viability in response to treatment of patient-derived GBM cell lines with **A**, **B**, bortezomib, **C**, **D**, everolimus, **E**, **F**, celecoxib, and **G**, **H**, pitavastatin. All drugs were tested *in vitro* at 1 μM for 72 h and viability was assayed using Alamar Blue assay. Cells showing decrease in viability of 20% or greater are considered sensitive to the drug.

We tested ten anti-cancer drugs *in silico* on the simulation avatars of the 8 patient-derived GBM cell lines in a blinded prospective study. These simulations generated predictions that we compared with *in vitro* experimental data (Additional file 1: Table S7A-D). Of the 80 *in silico* predictions, 61 (76.25%) predictions showed agreement with *in vitro* experimental results. Analysis of drug sensitivity correlation for all 8 GBM patient-derived cell lines, for all the 13 drugs is summarized in Additional file 1: Table S7. Figures 5A-H and 6A-H show a drug-wise comparison of *in silico* predictions (dose-response curves) and *in vitro* experimental results generated with testing 1 μM concentration of each drug on these cell lines.

### Effect of tyrosine kinase inhibitors on patient-derived GBM cells

For the EGFR family inhibitor lapatinib, simulation studies predicted SK429, SK748 and SK1035 to be resistant, which were confirmed by *in vitro* data. Similarly, modeling predicted GBM8, SK102, SK262 and SK987 to be sensitive and these predictions were in agreement with experimental data (Figure 5A and B). However, modeling predicted GBM4 to be resistant to lapatinib while *in vitro* data showed GBM4 to be highly sensitive to lapatinib (Figure 5B). For the tyrosine kinase inhibitor nilotinib, the model predicted GBM8 to be sensitive while all the other profiles to be resistant (Figure 5C). *In vitro* studies demonstrated that GBM8 was indeed sensitive to nilotinib as predicted, but there was a mismatch with the experimental results for two lines – SK262 and SK1035. Experimentally, SK262 was found to be sensitive, whereas SK1035 was on the borderline of sensitivity and resistance (Figure 5D). For imatinib, simulation predicted that all GBM lines except GBM8 were resistant (Figure 5E). The experimental results corroborated with this *in silico* prediction (Figure 5F). Sunitinib was the other multi-tyrosine kinase inhibitor tested. Our simulation predicted GBM8, SK102 and SK987 to be sensitive to sunitinib; however, only GBM8 was found to be sensitive *in vitro*. SK262 was predicted to be resistant to sunitinib but *in vitro* data found it to be moderately sensitive. On the other hand, GBM4, SK429, SK748 and SK1035 were found to be resistant in both simulation and experimental data (Figure 5G-H).

### Effect of other drugs on patient-derived GBM cells

Besides the tyrosine kinase inhibitors, correlation between *in silico* predictions and experimental results for the 8 patient-derived GBM cell lines was also tested for drugs such as pitavastatin (HMG CoA reductase inhibitor), everolimus (mTOR inhibitor), celecoxib (COX2 inhibitor) and bortezomib (proteasome inhibitor) (Figure 6 A-H). For bortezomib, all profiles were predicted to be sensitive and these predictions matched with *in vitro* experimental results (Figure 6A and B). For everolimus, *in vitro* results were in agreement with simulation predictions for all lines except SK429 (Figure 6C and D). Our *in silico* model predicted GBM4, SK262, SK429, SK748 and SK1035 to be resistant to celecoxib; these predictions matched with *in vitro* results. However, GBM8, SK102 and SK987 were predicted to show moderate sensitivity to celecoxib, but were found to be resistant *in vitro* (Figure 6E and F). For pitavastatin, the simulation predicted 5 patient-derived GBM cell lines to be sensitive (GBM8, GBM4, SK102, SK262 and SK987), of which SK987 was found to be resistant *in vitro*. On the other hand, of the cell lines predicted to be resistant (SK429, SK748 and SK1035), SK1035 was sensitive *in vitro* and did not match with the prediction (Figure 6G and H).

These data demonstrate a 76.25% agreement between *in silico* predictions of drug response and *in vitro* experimental data in patient-derived GBM cell lines.

## Discussion

Developing an *in silico* model that takes into account the complex genotypes/phenotypes of cancer to accurately predict drug response will help personalize therapy with more efficiency. In this study, we developed and validated a virtual tumor model by retrospectively testing it against a dataset from a recent screening study [23]; we obtained a corroboration of ~85% between our predictions and the results from this study. Following this retrospective validation, we generated *in silico* predictions to prospectively test the sensitivity of patient-derived GBM cell lines to targeted agents. These analyses also demonstrated a high degree of agreement (>75%) between *in vitro* experimental findings and *in silico* predictions. These studies validate our *in silico* tumor model and the simulation-based approach and provide critical proof-of-concept of *a priori* prediction of responses to targeted therapies. Thus, this model provides an effective platform for testing and developing personalized therapeutic regimens for cancer patients.

The genomic inputs that we used to create simulation avatars for patient-derived GBM cell lines were copy number variation data. A more comprehensive and accurate profile would require additional data (gene mutations, methylation status etc. along with copy number variation); this would help us develop a more representative avatar and would likely improve the accuracy of our drug response predictions and provide higher correlation with experimental data.

Genotypes of cancer cell lines have traditionally been used to correlate with drug sensitivity [28,29]. A similar recent study makes efficient use of gene expression profiles to categorize colorectal cancers into different molecular and clinically actionable subtypes [30]. Moreover, it is clear that using molecular tumor profiles to stratify patients for therapy affects response and progression-free survival [31]. However, increasing amounts of data from genomic, proteomic, transcriptomic and metabolomic profiling will likely require integration of these varied datasets and development of predictive systems modeling, which may hold the key to effective cancer therapy.

Rapid screening of patient samples in real time with models such as the one we have developed can drive critical therapeutic decision-making. Although our current model makes only cell-intrinsic predictions, we have been able to achieve a high rate of agreement between *in silico* predictions and *in vitro* findings. Future versions of this model are being refined to incorporate tumor microenvironment including aspects of angiogenesis, hypoxia, and tumor-associated inflammation. We believe that incorporating these features into our model would more accurately represent the tumor in a patient. Importantly, this will further help improve our predictions for designing therapeutic regimens for GBM patients. This model can also be adapted to identify potential mechanisms of resistance *a priori* and to design rational drug combinations that prevent emergence of resistance and development of escape pathways.

Our *in silico* model aligns with NCI guidelines that emphasize evaluation of similar predictor models to determine their accuracy [12,15,32,33]. We intend to test this model in clinical trials and utilize it as a tool to expedite clinical decision-making and determine drugs/combinations most likely to benefit a patient. Additionally, models such as these will play important roles in testing new biological hypotheses. This is critical to the discovery of molecular drivers and critical networks in cancer pathophysiology and the development of better diagnostics and effective therapeutics.

## Competing interests

The following authors are employed by Cellworks, Inc.: Zeba Sultana, Taher Abbasi, Shweta Kapoor, Ansu Kumar, Shahabuddin Usmani, Ashish Agrawal, and Shireen Vali. The other authors report no competing financial interests.

## Authors’ contributions

SCP – designed study, performed research, analyzed data, wrote manuscript; ZS – executed simulation studies, analyzed data, wrote manuscript; SP – designed study, performed research, analyzed data, wrote manuscript; PJ – performed research, analyzed data; RM – performed research, analyzed data; YC – performed research, analyzed data; ISB – performed research; NN – performed research; MM – performed research, analyzed data; TA – analyzed data, developed analytics, wrote manuscript; SK – developed predictive simulation-based tumor cell technology; AK – developed predictive simulation-based tumor cell technology; SU – executed simulation studies, developed predictive simulation-based tumor cell technology; AA – developed predictive simulation-based tumor cell technology; SV – analyzed data, developed predictive simulation-based tumor cell technology, developed analytics, wrote manuscript; SK (Santosh Kesari) – designed study, planned and directed research, analyzed data, wrote manuscript. All authors read and approved the final manuscript.

## Supplementary Material

Additional file 1 Supplementary Information. Click here for file

## References

[B1] Wen PY Kesari S Malignant gliomas in adults N Engl J Med 2008 359 492 507 10.1056/NEJMra0708126 18669428

[B2] Mischel PS Shai R Shi T Horvath S Lu KV Choe G Seligson D Kremen TJ Palotie A Liau LM Cloughesy TF Nelson SF Identification of molecular subtypes of glioblastoma by gene expression profiling Oncogene 2003 22 2361 2373 10.1038/sj.onc.1206344 12700671

[B3] TCGA Comprehensive genomic characterization defines human glioblastoma genes and core pathways Nature 2008 455 1061 1068 10.1038/nature07385 18772890 PMC2671642

[B4] Verhaak RG Hoadley KA Purdom E Wang V Qi Y Wilkerson MD Miller CR Ding L Golub T Mesirov JP Alexe G Lawrence M O'Kelly M Tamayo P Weir BA Gabriel S Winckler W Gupta S Jakkula L Feiler HS Hodgson JG James CD Sarkaria JN Brennan C Kahn A Spellman PT Wilson RK Speed TP Gray JW Meyerson M Integrated genomic analysis identifies clinically relevant subtypes of glioblastoma characterized by abnormalities in PDGFRA, IDH1, EGFR, and NF1 Cancer Cell 2010 17 98 110 10.1016/j.ccr.2009.12.020 20129251 PMC2818769

[B5] Deisboeck TS Personalizing medicine: a systems biology perspective Mol Syst Biol 2009 5 249 19293829 10.1038/msb.2009.8 PMC2671924

[B6] Deisboeck TS Zhang L Yoon J Costa J *In silico* cancer modeling: is it ready for prime time? Nat Clin Pract Oncol 2009 6 34 42 10.1038/ncponc1237 18852721 PMC3212937

[B7] Woodward DE Cook J Tracqui P Cruywagen GC Murray JD Alvord EC Jr A mathematical model of glioma growth: the effect of extent of surgical resection Cell Prolif 1996 29 269 288 10.1111/j.1365-2184.1996.tb01580.x 8809120

[B8] Tracqui P Cruywagen GC Woodward DE Bartoo GT Murray JD Alvord EC Jr A mathematical model of glioma growth: the effect of chemotherapy on spatio-temporal growth Cell Prolif 1995 28 17 31 10.1111/j.1365-2184.1995.tb00036.x 7833383

[B9] Zhang L Wang Z Sagotsky JA Deisboeck TS Multiscale agent-based cancer modeling J Math Biol 2009 58 545 559 10.1007/s00285-008-0211-1 18787828

[B10] Deisboeck TS Wang Z Macklin P Cristini V Multiscale cancer modeling Annu Rev Biomed Eng 2011 13 127 155 10.1146/annurev-bioeng-071910-124729 21529163 PMC3883359

[B11] Almine JF Wise SG Hiob M Singh NK Tiwari KK Vali S Abbasi T Weiss AS Elastin sequences trigger transient proinflammatory responses by human dermal fibroblasts FASEB J 2013 27 3455 3465 10.1096/fj.13-231787 23671273 PMC3752536

[B12] Barve A Gupta A Solapure SM Kumar A Ramachandran V Seshadri K Vali S Datta S A kinetic platform for *in silico* modeling of the metabolic dynamics in Escherichia coli Adv Appl Bioinform Chem 2010 3 97 110 21918631 10.2147/AABC.S14368 PMC3170011

[B13] Cirstea D Hideshima T Rodig S Santo L Pozzi S Vallet S Ikeda H Perrone G Gorgun G Patel K Desai N Sportelli P Kapoor S Vali S Mukherjee S Munshi NC Anderson KC Raje N Dual inhibition of akt/mammalian target of rapamycin pathway by nanoparticle albumin-bound-rapamycin and perifosine induces antitumor activity in multiple myeloma Mol Cancer Ther 2010 9 963 975 10.1158/1535-7163.MCT-09-0763 20371718 PMC3096071

[B14] Equils O Nambiar P Hobel CJ Smith R Simmons CF Vali S A computer simulation of progesterone and Cox2 inhibitor treatment for preterm labor PLoS One 2010 5 e8502 10.1371/journal.pone.0008502 20111699 PMC2811723

[B15] Harvey LE Kohlgraf KG Mehalick LA Raina M Recker EN Radhakrishnan S Prasad SA Vidva R Progulske-Fox A Cavanaugh JE Vali S Brogden KA Defensin DEFB103 bidirectionally regulates chemokine and cytokine responses to a pro-inflammatory stimulus Sci Rep 2013 3 1232 23390582 10.1038/srep01232 PMC3565171

[B16] Kannaiyan R Hay HS Rajendran P Li F Shanmugam MK Vali S Abbasi T Kapoor S Sharma A Kumar AP Chng WJ Sethi G Celastrol inhibits proliferation and induces chemosensitization through down-regulation of NF-kappaB and STAT3 regulated gene products in multiple myeloma cells Br J Pharmacol 2011 164 1506 1521 10.1111/j.1476-5381.2011.01449.x 21506956 PMC3221104

[B17] Kaushik P Gorin F Vali S Dynamics of tyrosine hydroxylase mediated regulation of dopamine synthesis J Comput Neurosci 2007 22 147 160 10.1007/s10827-006-0004-8 17053993

[B18] Tandon R Kapoor S Vali S Senthil V Nithya D Venkataramanan R Sharma A Talwadkar A Ray A Bhatnagar PK Dastidar SG Dual epidermal growth factor receptor (EGFR)/insulin-like growth factor-1 receptor (IGF-1R) inhibitor: a novel approach for overcoming resistance in anticancer treatment Eur J Pharmacol 2011 667 56 65 10.1016/j.ejphar.2011.04.066 21640718

[B19] Vali S Mythri RB Jagatha B Padiadpu J Ramanujan KS Andersen JK Gorin F Bharath MM Integrating glutathione metabolism and mitochondrial dysfunction with implications for Parkinson's disease: a dynamic model Neuroscience 2007 149 917 930 10.1016/j.neuroscience.2007.08.028 17936517

[B20] Vali S Pallavi R Kapoor S Tatu U Virtual prototyping study shows increased ATPase activity of Hsp90 to be the key determinant of cancer phenotype Syst Synth Biol 2010 4 25 33 10.1007/s11693-009-9046-3 19856130 PMC2816227

[B21] Galli R Binda E Orfanelli U Cipelletti B Gritti A De Vitis S Fiocco R Foroni C Dimeco F Vescovi A Isolation and characterization of tumorigenic, stem-like neural precursors from human glioblastoma Cancer Res 2004 64 7011 7021 10.1158/0008-5472.CAN-04-1364 15466194

[B22] Lee J Kotliarova S Kotliarov Y Li A Su Q Donin NM Pastorino S Purow BW Christopher N Zhang W Park JK Fine HA Tumor stem cells derived from glioblastomas cultured in bFGF and EGF more closely mirror the phenotype and genotype of primary tumors than do serum-cultured cell lines Cancer Cell 2006 9 391 403 10.1016/j.ccr.2006.03.030 16697959

[B23] Garnett MJ Edelman EJ Heidorn SJ Greenman CD Dastur A Lau KW Greninger P Thompson IR Luo X Soares J Liu Q Iorio F Surdez D Chen L Milano RJ Bignell GR Tam AT Davies H Stevenson JA Barthorpe S Lutz SR Kogera F Lawrence K McLaren-Douglas A Mitropoulos X Mironenko T Thi H Richardson L Zhou W Jewitt F Systematic identification of genomic markers of drug sensitivity in cancer cells Nature 2012 483 570 575 10.1038/nature11005 22460902 PMC3349233

[B24] Curtin JA Fridlyand J Kageshita T Patel HN Busam KJ Kutzner H Cho KH Aiba S Brocker EB LeBoit PE Pinkel D Bastian BC Distinct sets of genetic alterations in melanoma N Engl J Med 2005 353 2135 2147 10.1056/NEJMoa050092 16291983

[B25] Davies H Bignell GR Cox C Stephens P Edkins S Clegg S Teague J Woffendin H Garnett MJ Bottomley W Davis N Dicks E Ewing R Floyd Y Gray K Hall S Hawes R Hughes J Kosmidou V Menzies A Mould C Parker A Stevens C Watt S Hooper S Wilson R Jayatilake H Gusterson BA Cooper C Shipley J Mutations of the BRAF gene in human cancer Nature 2002 417 949 954 10.1038/nature00766 12068308

[B26] Konecny GE Pegram MD Venkatesan N Finn R Yang G Rahmeh M Untch M Rusnak DW Spehar G Mullin RJ Keith BR Gilmer TM Berger M Podratz KC Slamon DJ Activity of the dual kinase inhibitor lapatinib (GW572016) against HER-2-overexpressing and trastuzumab-treated breast cancer cells Cancer Res 2006 66 1630 1639 10.1158/0008-5472.CAN-05-1182 16452222

[B27] Yost SE Pastorino S Rozenzhak S Smith EN Chao YS Jiang P Kesari S Frazer KA Harismendy O High-resolution mutational profiling suggests the genetic validity of glioblastoma patient-derived pre-clinical models PLoS One 2013 8 e56185 10.1371/journal.pone.0056185 23441165 PMC3575368

[B28] Shoemaker RH Monks A Alley MC Scudiero DA Fine DL McLemore TL Abbott BJ Paull KD Mayo JG Boyd MR Development of human tumor cell line panels for use in disease-oriented drug screening Prog Clin Biol Res 1988 276 265 286 3051021

[B29] Weinstein JN Myers TG O'Connor PM Friend SH Fornace AJ Jr Kohn KW Fojo T Bates SE Rubinstein LV Anderson NL Buolamwini JK van Osdol WW Monks AP Scudiero DA Sausville EA Zaharevitz DW Bunow B Viswanadhan VN Johnson GS Wittes RE Paull KD An information-intensive approach to the molecular pharmacology of cancer Science 1997 275 343 349 10.1126/science.275.5298.343 8994024

[B30] Sadanandam A Lyssiotis CA Homicsko K Collisson EA Gibb WJ Wullschleger S Ostos LC Lannon WA Grotzinger C Del Rio M Lhermitte B Olshen AB Wiedenmann B Cantley LC Gray JW Hanahan D A colorectal cancer classification system that associates cellular phenotype and responses to therapy Nat Med 2013 19 619 625 10.1038/nm.3175 23584089 PMC3774607

[B31] Von Hoff DD Stephenson JJ Jr Rosen P Loesch DM Borad MJ Anthony S Jameson G Brown S Cantafio N Richards DA Fitch TR Wasserman E Fernandez C Green S Sutherland W Bittner M Alarcon A Mallery D Penny R Pilot study using molecular profiling of patients’ tumors to find potential targets and select treatments for their refractory cancers J Clin Oncol 2010 28 4877 4883 10.1200/JCO.2009.26.5983 20921468

[B32] Rajendran P Ong TH Chen L Li F Shanmugam MK Vali S Abbasi T Kapoor S Sharma A Kumar AP Hui KM Sethi G Suppression of signal transducer and activator of transcription 3 activation by butein inhibits growth of human hepatocellular carcinoma in vivo Clin Cancer Res 2011 17 1425 1439 10.1158/1078-0432.CCR-10-1123 21131551

[B33] Sultana Z Paleologou KE Al-Mansoori KM Ardah MT Singh N Usmani S Jiao H Martin FL Bharath MM Vali S El-Agnaf OM Dynamic modeling of alpha-synuclein aggregation in dopaminergic neuronal system indicates points of neuroprotective intervention: experimental validation with implications for Parkinson's therapy Neuroscience 2011 199 303 317 22056602 10.1016/j.neuroscience.2011.10.018

