# Supplementary material for: In silico modeling predicts drug sensitivity of patient-derived cancer cells
Source: J Transl Med. 2014 May 21;12:128. doi: 10.1186/1479-5876-12-128 (PMC4030016; doi:10.1186/1479-5876-12-128)
Supplement: Additional file 1 — Supplementary Information. [file 1479-5876-12-128-S1.doc]

**Supplementary Information**

*In Silico* Modeling Predicts Drug Sensitivity of Patient-Derived Cancer Cells

Sandeep C. Pingle^1†^, Zeba Sultana^2†^, Sandra Pastorino^1^, Pengfei Jiang^1^, Rajesh Mukthavaram^1^, Ying Chao^1^, Ila Sri Bharati^1^, Natsuko Nomura^1^, Milan Makale^1^, Taher Abbasi^3^, Shweta Kapoor^2^, Ansu Kumar^2^, Shahabuddin Usmani^2^, Ashish Agrawal^2^, Shireen Vali^2,3^, Santosh Kesari^1,4^*

**Affiliations:**

^1^Translational Neuro-Oncology Laboratories, Moores Cancer Center, UC San Diego, La Jolla, CA 92093

^2^Cellworks Research India Ltd., Bangalore, India 560 066

^3^Cellworks Group Inc., Saratoga, CA 95070

^4^Department of Neurosciences, UC San Diego, La Jolla, CA 92093

† These authors contributed equally to this work.

*Corresponding author: Santosh Kesari (skesari@ucsd.edu)

1. ***In Silico* Tumor Model – Overview**

The simulation experiments and analyses were performed using the predictive tumor model, a comprehensive and dynamic representation of signaling and metabolic pathways in the context of cancer physiology. The simulation model includes representation of important signaling pathways implicated in cancer such as growth factors like EGFR, PDGFR, FGFR, c-MET, VEGFR and IGF-1R; cytokine and chemokines like IL1, IL4, IL6, IL12, TNF; GPCR mediated signaling pathways; mTOR signaling; cell cycle regulations, tumor metabolism, oxidative and ER stress, representation of autophagy and proteosomal degradation, DNA damage repair, p53 signaling and apoptotic cascade. The referenced current version of cancer model includes more than 4700 intracellular biological entities and ~6500 reactions representing their interactions regulated by ~25000 kinetic parameters. This comprises a comprehensive and extensive coverage of the kinome, transcriptome, proteome and the metabolome. There are 142 kinases and 102 transcription factors modeled in the system.

1. **Procedure for Model Development**

A bottom-up approach was used for building the complete model; the different pathways were developed as individual blocks and then sequentially integrated to get the final tumor cell model. A signaling pathway is the series of cellular events that are triggered by the activating ligand. Each of these cellular events, is represented by reaction nodes where biological entities (like receptors, adaptor proteins, enzymes, transcription factors, mRNA, metabolites etc.) act as substrates/input to a reaction and give the transformed (such as dimerized, phosphorylated or cleaved) entity as output. The biological entities can also act as modulators (activators/inhibitors) of a reaction. The conversion of the substrates into products is governed by mathematical equations (such as Michaelis-Menton or mass action equations) defined in the reaction node. In cases where experimentally derived kinetic parameters for these equations are unavailable, some experimentally reported results from published literature are used as alignment studies to reverse engineer and derive the kinetic parameters. During simulation the time-dependent changes in these reaction fluxes are solved in the form of ordinary differential equations (ODE).

Every reaction is validated by testing the sensitivity of the various parameters of the reaction to match with the known regulatory mechanisms and experimental results. Upon integration of the complete model, the predictive results from the *in silico* model are validated against an extensive set of experimental results. The procedure for model development is illustrated below in further details by taking one of the modules from the integrated system, the EGFR pathway as an example.

**Illustration of Pathway Development in Simulation Model – EGFR**

The major signaling pathways downstream to EGFR activation have been studied and reviewed extensively in the literature [1]; we illustrate this signaling pathway in **Supplementary Figure 1**. A snapshot of the representation of these signaling events in the model is further illustrated in **Supplementary Figure 2** using ~30 reaction nodes. In the cell, the process of signaling and regulations involve physical binding of substrate proteins and their modulators. These have been represented as activations or inhibitions capturing their functional impact in the predictive simulation model. The flux equations of these reaction nodes have been listed here in Supplementary Information in **Box 1**.

Binding of extracellular domain of EGFR to its ligands such as EGF (used in this example) induces receptor dimerization/activation of intrinsic tyrosine kinase activity and autophosphorylation – this is represented in the reaction nodes 1 and 2 in **Supplementary Figure 2** and **Box 1**. Tyrosine phosphorylation of EGFR leads to the recruitment of diverse signaling proteins, including adaptor proteins like GRB2 (Growth Factor Receptor-Bound Protein-2), SHC (Src Homology-2 Domain Containing Transforming Protein), PLC-Gamma (phospholipase C gamma), STAT (Signal Transducer and Activator of Transcription), and several other molecules. These have been represented in subsequent reaction nodes.

The SH2 domain of GRB2 can bind directly to phosphotyrosines 1068 and 1086 of the activated EGFR or indirectly through the tyrosine-phosphorylated adaptor protein SHC and activate SOS, an exchange factor of RasGTPase. Node numbers 3, 4 and 5 in the illustration represent these activation events.

SOS is placed in proximity to Ras by the binding of the GRB2 and SOS complex to EGFR, and leads to GTP-loading of Ras (reaction node 6) with subsequent activation of Ras effectors, such as Raf kinases (reaction node 7) and PI3K (reaction node 8). Raf activation triggers a cascade of phosphorylation events including the phosphorylation and activation of the MEK (reaction nodes 9, 10) followed by ERK (reaction nodes 11, 12). Phosphorylated ERK causes inhibition of many of the upstream reaction nodes to form a negative feedback loop that plays a major role in maintaining homeostasis in the living cell. Phosphorylated ERK inhibits Ras-GTP mediated activation of Raf (reaction node 7), phosphorylated GRB2-mediated activation of SOS1 (reaction node 5) and GAB1 (reaction node 13). Apart from activation of PI3K by Ras-GTP, the docking protein GAB1 can bind to phosphorylated GRB2 (reaction node 13) and thus mediate EGFR-induced PI3K stimulation.

Activated PI3K phosphorylates membrane bound PIP2 to generate PIP3 (reaction node 14). PIP3, by binding to the PH domain of AKT anchors it to the plasma membrane and facilitates co-localization of PDPK1 and AKT (reaction nodes 15 and 16). Consequently, AKT is phosphorylated at Thr308 by PDPK1 (reaction node 18). A priming phosphorylation of AKT at serine 473 is carried out prior to this by mTORC2 (reaction node 17). A negative feedback from the Ras🡪Raf 🡪ERK signaling axis at this phosphorylation is inhibition by V600E BRAF mutant. Activated AKT then phosphorylates several substrates that signal cell survival and proliferation. One of the major downstream effectors of AKT is the mTOR kinase.

TSC1 and TSC2 are tumor suppressor genes that are negative regulators of the mTOR-S6K pathway. AKT-mediated serine phosphorylation of TSC1/2 complex causes inhibition of the tumor suppressor function of TSC1/2 (reaction node 19). On the other hand, threonine phosphorylation of TSC1/2 by the energy-sensing kinase AMPK (reaction node 20) activates its mTOR-suppressive function. Unphosphorylated TSC1/2 complex causes basal level of conversion of RHEB_GTP to RHEB_GDP (reaction node 21). However, TSC1/2 phosphorylated at Thr 2446 by AMPK causes inactivation of RHEB_GTP to RHEB_GDP (reaction node 22). RHEB_GDP can be recycled to an activated state (reaction node 23). RHEB_GTP activates the mTOR complex1 (reaction node 24), which directly supports cell growth via the S6K and 4EBP pathways that regulate translation.

One of the prominent enzymes activated by EGFR is PLCgamma1 (reaction node 25). This enzyme has two SH2 domains and can catalyze the hydrolysis of PIP2 to generate the second messengers DAG and IP3 (reaction node 26). IP3 diffuses through the cytosol and releases stored Ca^2+^ ions from the ER. DAG is the physiological activator of PKC (reaction node 27), which in turn leads to phosphorylation of various substrate proteins.

Other important signaling events by activated EGFR include Tyrosine phosphorylation of STAT1 and STAT3, secondary to formation of complex of STAT1/STAT3 with JAK1/JAK2. This has been represented by reaction nodes 28 and 29 showing phosphorylation of JAK1 and JAK2. This complex has further been shown to cause Tyrosine phosphorylation of STAT1 (reaction node 30) and STAT3 (reaction node 31) that dimerize to form active transcription factors that translocate to nucleus and regulate expression of genes implicated in tumor growth.

**Box 1: Reactions and Equations in EGFR signaling, as represented in the *in silico* tumor model**.

| **Node** | **Reaction** | **Mechanism** | **Reaction Equation** | **References**  **(PubMed ID)** |
| --- | --- | --- | --- | --- |
| **1** | Binding of EGF ligand with EGF receptor  **[EGFR_f  EGFR_di]**  Binding of the ligand induces dimerization of the EGF receptor | The reaction is modeled as a Simple Michaelis-Menten equation, with receptor as the substrate and ligand as the activator. ERKpp is an inhibitor of this reaction, which is experimentally reported negative-feedback loop. | (Vf_EGF*(EGFR_f.Concentration*EGFR_f.Concentration))/((Km_EGFR_f+EGFR_f.Concentration)*(Km_EGFR_f+EGFR_f.Concentration))  Where,  Vf_EGF=(kcatf_EGF*EGF_ec.Concentration)*VCyt)/ Ki_MAPK3_MAPK1_pp_app | **11531336, 22552284** |
| **2** | Autophosphorylation of EGFR homodimer  **[EGFR_di  EGFR_di_p]**  Ligand binding induces dimerization, activation of intrinsic tyrosine kinase activity of EGFR | The reaction is modeled as a Simple Michaelis-Menten equation that is auto-catalyzed. | (Vf*EGFR_di.Concentration)/( Km_EGFR_di +EGFR_di.Concentration) | **12134089** |
| **3** | Binding and Phosphorylation of SHC1 by EGF receptor  **[SHC1  SHC1_p]**  SHC1: an adaptor protein that gets tyrosine phosphorylated by activated EGFR | The reaction is modeled as a Simple Michaelis-Menten equation, with phosphorylated EGFR homodimer as an activator. | ((Vf_EGFR_di_p*SHC1.Concentration)/(Km_SHC1+SHC1.Concentration))  Where,  Vf_EGFR_di_p = (kcatf_EGFR_di_p *EGFR_di_p. Concentration)*VCyt | **9544989** |
| **4** | Binding and Phosphorylation of GRB2 by EGF receptor  **[GRB2  GRB2_p]**  The SH2 domain of GRB2 can directly bind to pTyr 1068 and 1086 of activated EGFR and get phosphorylated.  This is in addition to SHC-mediated Grb2 activation. | The reaction is modeled as a Simple Michaelis-Menten equation with phosphorylated EGFR homodimer as an activator. | ((Vf_EGFR_di_p*GRB2.Concentration)/(Km_GRB2+GRB2.Concentration))  Where,  Vf_EGFR_di_p = (kcatf_EGFR_di_p *EGFR_di_p. Concentration)*VCyt | **7527043** |
| **5** | Binding and Phosphorylation of SOS1 by GRB2  **[SOS1  SOS1_act]**  SOS1 is a Ras GEF that gets activated by GRB2p**.** This activation of SOS1 is inhibited by phosphorylated ERK. | The reaction is modeled as a Simple Michaelis-Menten equation with GRB2 as an activator.  The reaction flux also incorporates inhibition by activated ERK, which is a negative feedback loop in this signaling axis. | ((Vf_GRB2_p_app*SOS1.Concentration)/(Km_SOS1+SOS1.Concentration))  Where,  Vf_GRB2_p_app = (Vf_GRB2_p/(1+(MAPK3_MAPK1_pp.Concentration/Ki_MAPK3_MAPK1_pp)))  Vf_GRB2_p = (kcatf_GRB2_p*GRB2_p.Concentration)*VCyt | **7592690** |
| **6** | Activation of RAS by EGFR activated SOS1  **[RAS_GDP  RAS_GTP]** | The reaction is modeled as a Simple Michaelis-Menten equation with SOS1 as an activator | ((Vf_SOS1_act*RAS_GDP.Concentration)/(Km_RAS_GDP+RAS_GDP.Concentration)) Where,  Vf_SOS1_act = ((kcatf_SOS1_act*SOS1_act.Concentration)*VCyt) | **7592690** |
| **7** | Phosphorylation of RAF by RAS-GTP  **[RAF  RAF_p]**  RAS_GTP phosphorylates RAF kinase, one of its effector proteins. This reaction is inhibited by pERK, which is a negative feedback loop of Ras🡪Raf🡪ERK signaling axis. Additionally, it is also inhibited by pAKT, a well-reported example of cross-talks between different signaling networks in a living cell. | The reaction is modeled as a Simple Michaelis-Menten equation with RAS-GTP as an activator.  It also incorporates negative feedback inhibition by ERK and AKT. | ((Vf_RAS_GTP_app*RAF.Concentration)/(Km_RAF+RAF.Concentration))  Where,  Vf_RAS_GTP_app=  (Vf_RAS_GTP/(1+(MAPK3_MAPK1_pp.Concentration/Ki_MAPK3_MAPK1_pp)+(AKT1_pp.Concentration/Ki_AKT1_pp))  and,  Vf_RAS_GTP= ((kcatf_RAS_GTP*RAS_GTP. Concentration)*VCyt) | **8307946** |
| **8** | Activation of PI3K by EGFR activated GAB1 and by RAS-GTP  **[PIK3CA  PIK3CA_act]** | The reaction is modeled as a Simple Michaelis-Menten equation with GAB1 as one activator and RAS_GTP as the other independent activator. | ((Vf_GAB1_P*PIK3CA.Concentration)/(Km_PIK3CA+PIK3CA.Concentration)) + ((Vf_RAS_GTP*PIK3CA.Concentration)/(Km_PIK3CA+PIK3CA.Concentration))  Where,  Vf_GAB1_P= ((kcatf_GAB1_P*GAB1_P. Concentration)*VCyt) and  Vf_RAS_GTP= ((kcatf_RAS_GTP*RAS_GTP. Concentration)*VCyt) | **15550174** |
| **9** | Phosphorylation and activation of MEK by RAF  **[MAP2K1_MAP2K2  MAP2K1_MAP2K2_p]** | The reaction is modeled as a Simple Michaelis-Menten equation with RAF as an activator. | ((Vf_RAF_p*MAP2K1_MAP2K2.Concentration)/(Km_MAP2K1_MAP2K2+MAP2K1_MAP2K2.Concentration))  Where,  Vf_RAF_p= ((kcatf_RAF_p*RAF_p. Concentration)*VCyt) | **8019005** |
| **10** | Phosphorylation and activation of MEK by RAF  **[MAP2K1_MAP2K2_p  MAP2K1_MAP2K2_pp]** | The reaction is modeled as a Simple Michaelis-Menten equation with RAF as an activator. | ((Vf_RAF_p*MAP2K1_MAP2K2_p.Concentration)/(Km_MAP2K1_MAP2K2_p+MAP2K1_MAP2K2_p.Concentration))  Where,  Vf_RAF_p= ((kcatf_RAF*RAF_p. Concentration)*VCyt) | **8019005** |
| **11** | Phosphorylation and activation of ERK by MEK  **[MAPK3_MAPK1  MAPK3_MAPK1_p]** | The reaction is modeled as a Simple Michaelis-Menten equation with MEK as an activator. | ((Vf_MAP2K1_MAP2K2_pp*MAPK3_MAPK1.Concentration)/(Km_MAPK3_MAPK1+MAPK3_MAPK1.Concentration))  Where,  Vf_MAP2K1_MAP2K2_pp= ((kcatf_MAP2K1_MAP2K2_pp*MAP2K1_MAP2K2_pp. Concentration)*VCyt) | **8019005** |
| **12** | Phosphorylation and activation of ERK by MEK  **[MAPK3_MAPK1_p  MAPK3_MAPK1_pp]** | The reaction is modeled as a Simple Michaelis-Menten equation with MEK as an activator. | ((Vf_MAP2K1_MAP2K2_pp*MAPK3_MAPK1_p.Concentration)/(Km_MAPK3_MAPK1_p+MAPK3_MAPK1_p.Concentration))  Where,  Vf_MAP2K1_MAP2K2_pp= ((kcatf_MAP2K1_MAP2K2_pp*MAP2K1_MAP2K2_pp. Concentration)*VCyt) | **8019005** |
| **13** | Binding & Phosphorylation of GAB1 by GRB2p  **[GAB1  GAB1_p]**  docking protein GAB1 binds phosphorylated GRB2 and gets activated | The reaction is modeled as a Simple Michaelis-Menten equation with phosphorylated GRB2 as an activator.  The reaction flux also incorporates inhibition by activated ERK, which is a negative feedback loop in this signaling axis. | (Vf_GRB2_p * GAB1.Concentration)/(Km_GAB1+GAB1.Concentration))  Where,  Vf_GRB2_p = (kcatf_GRB2_p*GRB2_p.Concentration)*VCyt  and the flux is divided by the factor (1+(MAPK3_MAPK1_pp.Concentration/Ki_MAPK3_MAPK1_pp) | **5550174** |
| **14** | PI3K mediated conversion of PIP2 to PIP3  **[PI45P2  PI345P3]** | The reaction is modeled as a Simple Michaelis-Menten equation with PI3K as an activator. | ((Vf_PIK3CA_act*PI45P2.Concentration)/(Km_PI45P2+PI45P2.Concentration))  Where,  Vf_PIK3CA_act= ((kcatf_PIK3CA_act*PIK3CA_act. Concentration)*VCyt) | **11882383** |
| **14b** | PTEN mediated conversion of PIP3 to PIP2  **[PI345P3  PI45P2]** | The reaction is modeled as a Simple Michaelis-Menten equation with PTEN as a phosphatase (activator). | ((Vf_PTEN*PI345P3.Concentration)/(Km_PI345P3+PI345P3.Concentration))  Where,  Vf_PTEN= ((kcatf_PTEN*PTEN. Concentration)*VCyt) | **11882383** |
| **15** | Binding of and activation PDK1 by PI345P3  **[PDPK1  PDPK1_f]** This node is a representative of the membrane localization of PDPK1 by PIP3 binding. | The reaction is modeled as a Simple Michaelis-Menten equation with PIP3 as an activator. | ((Vf_PI345P3*PDPK1.Concentration)/(Km_PDPK1+PDPK1.Concentration))  Where,  Vf_PI345P3= ((kcatf_PI345P3*PI345P3. Concentration)*VCyt) | **11882383** |
| **16** | Binding of and activation AKT1 by PI345P3  **[AKT1_inact  AKT1]**  This node is a representative of the membrane localization of AKT1 by PIP3 binding. | The reaction is modeled as a Simple Michaelis-Menten equation with PIP3 as an activator. | ((Vf_PI345P3*AKT1_inact.Concentration)/(Km_AKT1_inact+AKT1_inact.Concentration))  Where,  Vf_PI345P3= ((kcatf_PI345P3*PI345P3. Concentration)*VCyt) | **11882383** |
| **17** | Phosphorylation of AKT1 by mTOR-Rictor on S473  **[AKT1  AKT1_p]** mTORC2 mediated priming phosphorylation of AKT at serine 473 | The reaction is modeled as a Simple Michaelis-Menten equation with mTORC2 as an activator. | ((Vf_MTOR_MAPKAP1_PRR5_MLST8_RICTOR*AKT1.Concentration)/(Km_AKT1+AKT1.Concentration))  Where,  Vf_MTOR_MAPKAP1_PRR5_MLST8_RICTOR= ((kcatf_MTOR_MAPKAP1_PRR5_MLST8_RICTOR*MTOR_MAPKAP1_PRR5_MLST8_RICTOR. Concentration)*VCyt) | **11882383** |
| **18** | Second phosphorylation of AKT1p by PDK1 on Thr308  **[AKT1_p  AKT1_pp]** | The reaction is modeled as a Simple Michaelis-Menten equation with PDPK1 as an activator. | ((Vf_PDPK1_f*AKT1_p.Concentration)/(Km_AKT1_p+AKT1_p.Concentration))  Where,  Vf_PDPK1_f= ((kcatf_PDPK1_f*PDPK1_f. Concentration)*VCyt) | **11882383** |
| **19** | Phosphorylation and inactivation of TSC1/2 complex by AKT  **[TSC1_TSC2  TSC1_TSC2_lser939_p]**  Phosphorylation of the TSC1_TSC2 tumor suppressor complex by AKT1pp that causes its inactivation. | The reaction is modeled as a Simple Michaelis-Menten equation with AKT as an activator. | ((Vf_AKT1_pp*TSC1_TSC2.Concentration)/(Km_TSC1_TSC2+TSC1_TSC2.Concentration))  Where,  Vf_AKT1_pp= ((kcatf_AKT1_pp*AKT1_pp. Concentration)*VCyt) | **12867426** |
| **20** | Phosphorylation and activation of TSC1/2 complex by AMPK  **[TSC1_TSC2  TSC1_TSC2_lthr2446_p]**  Phosphorylation of the TSC1_TSC2 tumor suppressor complex by activated AMPK that causes its activation. | The reaction is modeled as a Simple Michaelis-Menten equation with AMPK as an activator. | ((Vf_PRKAA1_p*TSC1_TSC2.Concentration)/(Km_TSC1_TSC2+TSC1_TSC2.Concentration))  Where,  Vf_PRKAA1_p = ((kcatf_ PRKAA1_p * PRKAA1_p. Concentration)*VCyt) | **18439900,**  **18466115** |
| **21** | Dephosphorylation (inactivation) of RHEB GTP by unphosphorylated TSC complex  **[RHEB_GTP  RHEB_GDP]** | The reaction is modeled as a Simple Michaelis-Menten equation with unphosphorylated TSC complex as an activator. | ((Vf_TSC1_TSC2*RHEB_GTP.Concentration)/(Km_RHEB_GTP+RHEB_GTP.Concentration))  Where,  Vf_TSC1_TSC2= ((kcatf_TSC1_TSC2*TSC1_TSC2. Concentration)*VCyt) | **15854902** |
| **22** | Dephosphorylation (inactivation) of RHEB GTP by TSC complex phosphorylated by AMPK  **[RHEB_GTP  RHEB_GDP]** | The reaction has been modeled as a Simple Michaelis Menten equation with Thr phosphorylated TSC complex as an activator | ((Vf_TSC1_TSC2_lthr2446_p*RHEB_GTP.Concentration)/(Km_RHEB_GTP+RHEB_GTP.Concentration))  Where,  Vf_TSC1_TSC2_lthr2446_p= ((kcatf_TSC1_TSC2_lthr2446_p*TSC1_TSC2_lthr2446_p. Concentration)*VCyt) | **15854902** |
| **23** | Conversion of RHEB GDP to RHEB GTP  **[RHEB_GDP  RHEB_GTP]** | The reaction has been modeled as a Simple Michaelis Menten equation | ((Vf*RHEB_GDP.Concentration)/(Km_RHEB_GDP+RHEB_GDP.Concentration)) | **15854902** |
| **24** | Activation of mTOR complex  **[MLST8_MTOR_RPTOR  MTOR_RPTOR_MLST8_RHEB_GTP**] | The reaction has been modeled as a Simple Michaelis Menten equation with RHEB-GTP as an activator | ((Vf_RHEB_GTP*MLST8_MTOR_RPTOR.Concentration)/(Km_MLST8_MTOR_RPTOR+MLST8_MTOR_RPTOR.Concentration))  Where,  Vf_RHEB_GTP= ((kcatf_RHEB_GTP*RHEB_GTP. Concentration)*VCyt) | **15854902** |
| **25** | Phosphorylation of PLC gamma by the EGF receptor  **[PLCG1 PLCG1_p]** | The reaction has been modeled as a Simple Michaelis Menten equation with EGFR as an activator | (Vf_EGFR_di_p*PLCG1.Concentration)/(Km_PLCG1+PLCG1.Concentration)  Where,  Vf_ EGFR_di_p = ((kcatf_EGFR_di_p*EGFR_di_p.Concentration)*VCyt) | **10473558** |
| **26** | PIP2 Hydrolysis by PLC gamma  **[PI45P2  DAG + IP3]** | The reaction has been modeled as a Simple Michaelis Menten equation with PLCG as an activator and PIP2 as the substrate | (Vf_PLCG1_p*PI45P2.Concentration)/(Km_PI45P2+PI45P2.Concentration)  Where,  Vf_PLCG1_p = (kcatf_PLCG1_p*PLCG1_p.Concentration)*VCyt | **19204146** |
| **27** | Binding of PKC-Calcium complex with DAG  **[PRKCA_p_Ca  PRKCA_p_Ca_DAG]** | The reaction has been modeled as a Simple Michaelis Menten equation with DAG as an activator and PKC Calcium complex as the substrate | (Vf_DAG*PRKCA_p_Ca.Concentration)/(Km_PRKCAp_ca_c+PRKCA_p_Ca.Concentration)  Where,  Vf_DAG = ((DAG.Concentration*kcatf_DAG)*VCyt) | **16893971** |
| **28** | Binding & Phosphorylation of JAK1 by EGF receptor  **[JAK1 EGFR_di_p_JAK1_p]** | The reaction has been modeled as a Simple Michaelis Menten equation with EGFR as an activator | (Vf_EGFR_di_p*JAK1.Concentration)/(Km_JAK1+JAK1.Concentration) | **8942998** |
| **29** | Binding & Phosphorylation of JAK2 by EGF receptor activated JAK1 complex  **[JAK2  EGFR_di_p_JAK1_p_JAK2_p]** | The reaction has been modeled as a Simple Michaelis Menten equation with EGFR_JAK1p complex as an activator | (Vf_EGFR_di_p_JAK1_p *JAK2.Concentration)/(Km_JAK2+JAK2.Concentration) | **8942998** |
| **30** | Phosphorylation of STAT1 by the EGF receptor activated JAK1-JAK2 complex  **[STAT1  STAT1_p]** | The reaction has been modeled as a Simple Michaelis Menten equation with EGFR_JAK1_JAK2 complex as an activator and incorporates inhibition by SOCS1, SOCS3 and PIAS4. | (Vf_EGFR_di_p_JAK1_p_JAK2_p*STAT1.Concentration)/(Km_STAT1+STAT1.Concentration)  Where,  Vf_EGFR_di_p_JAK1_p_JAK2_p = (kcatf_EGFR_di_p_JAK1_p_JAK2_p*EGFR_Cyt.EGFR_di_p_JAK1_p_JAK2_p.Concentration)*VCyt/Ki_PIAS4_app/Ki_SOCS1_app/Ki_SOCS3_app | **9368330** |
| **31** | Phosphorylation of STAT3 by the EGF receptor activated JAK1-JAK2 complex  **[STAT3  STAT3_p]** | The reaction has been modeled as a Simple Michaelis Menten equation with EGFR_JAK1_JAK2 complex as an activator and incorporates inhibition by SOCS1, SOCS3 and PIAS3. | (Vf_EGFR_di_p_JAK1_p_JAK2_p_app*STAT3.Concentration)/(Km_STAT3 +STAT3.Concentration)  Where,  Vf_EGFR_di_p_JAK1_p_JAK2_p_app = (Vf_EGFR2P_JAK1_JAK2p/(1+(PIAS3.Concentration/Ki_PIAS3)))/Ki_STAT3_lSer727_p_app/Ki_SOCS1_app/Ki_SOCS3_app | **9368330, 8942998** |

**Phosphorylation of STAT3 by the EGF receptor activated JAK1-JAK2 complex**

We have explained the last reaction (reaction node 31) in further detail below, enlisting all modulators of STAT3 activation, the scientific publications that report these modulations, the flux equations used, and methodology for reverse engineering of kinetic parameters.

[STAT3 🡪 STAT3_p]


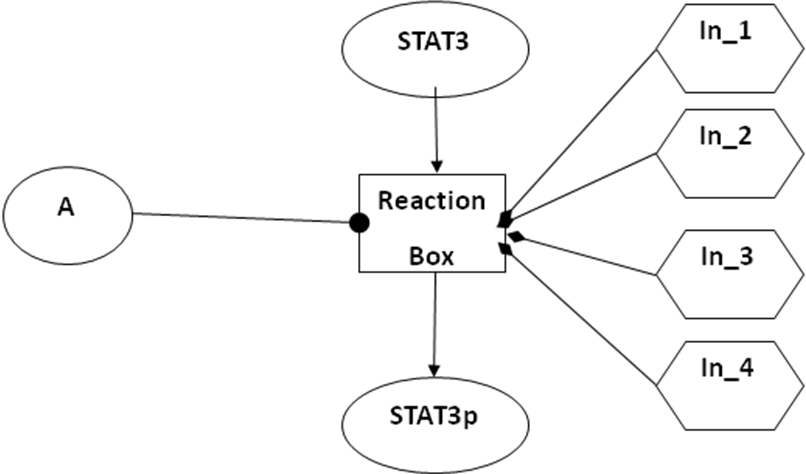


The reaction has been modeled as a simple Michaelis-Menten equation with:

Substrate – STAT3,

Product – STAT3_P,

Activator – A, and

Inhibited by 4 inhibitors – In_1, In_2, In_3, and In_4

A is the activated and dimerized EGFR receptor complex with JAK1 and JAK2 kinases.

The 4 inhibitors are:

• In_1 – PIAS3

• In_2 – SOCS1

• In_3 – SOCS3

• In_4 – STAT3 phosphorylated at Serine 727

The flux equation used is :

(Vf_A_app * STAT3 concentration) /( Km_STAT3 + STAT3.concentration).

Vf_A_app = Vf A / {1 + (In_1 concentration/Ki_In_1)+(In_2 concentration/Ki_In_2)+(In_3

concentration/Ki_In_3)+(In_4 concentration/Ki_In_4)}

where,

**Vf** defines the rate of the reaction. It is the product of Kcat (turn over number of the activator

driving the reaction) and the concentration of activator.

**Km** is the affinity of the substrate (low Km indicating a high affinity).

**Ki** of an inhibitor is the parameter that determines the extent of inhibitory influence of the inhibitor on the reaction. Mathematically, it is the concentration of the inhibitor required to inhibit the reaction by 50%.

**Box 2: Experimental Support for the Reaction Mechanism for STAT3 phosphorylation**

| **Mechanism** | **Description** | **Parameters** | | **Title (with reference)** | |
| --- | --- | --- | --- | --- | --- |
| Activation by A | EGFR-complex mediated activation of STAT3 | Concentration of A = Dynamic*  Kcat of A = 624 1/sec  Vf of A = Kcat*dynamic concentration of A  STAT3 Km = 6.70E-2 μM; Concentration of STAT3 = 0.3 μM | | In vitro activation of Stat3 by epidermal growth factor receptor kinase [2]  Jaks and Stats in cytokine signaling [3] | |
|  |  |  | |  | |
| Inhibition by In_1 | PIAS3 mediated inhibition of STAT3 -Tyr705 phosphorylation | Concentration of PIAS3 = 0.1 μM  Ki of PIAS3 = 0.1 μM | | Specific inhibition of Stat3 signal transduction by PIAS3 [4]  The association and nuclear translocation of the PIAS3-STAT3 complex is ligand and time dependent [5]  Protein inhibitor of activated STAT3 expression in lung cancer [6] | |
|  |  |  | |  | |
| Inhibition by In_2 | SOCS1 mediated inhibition of STAT3 -Tyr705 phosphorylation | Concentration of SOCS1 = Dynamic**  Ki of SOCS1 = 19 μM | | Anti-proliferative effect of SOCS-1 through the suppression of STAT3 and p38 MAPK activation in gastric cancer cells [7]  SOCS1 induced by NDRG2 expression negatively regulates STAT3 activation in breast cancer cells [8]  Epigenetic modification of SOCS-1 differentially regulates STAT3 activation in response to interleukin-6 receptor and epidermal growth factor receptor signaling through JAK and/or MEK in head and neck squamous cell carcinomas [9] | |
|  |  |  | |  | |
| Inhibition by In_3 | SOCS3 mediated inhibition of STAT3 -Tyr705 phosphorylation | Concentration of SOCS3 = Dynamic**  Ki of SOCS3 = 0.3 μM | | SOCS3 exerts its inhibitory function on interleukin-6 signal transduction through the SHP2 recruitment site of gp130 [10]  Suppressor of cytokine signaling 3 inhibits breast tumor kinase activation of STAT3 [11]  Platelet factor 4 induces cell apoptosis by inhibition of STAT3 via up-regulation of SOCS3 expression in multiple myeloma [12]  IFN gamma-dependent SOCS3 expression inhibits IL-6-induced STAT3 phosphorylation and differentially affects IL-6 mediated transcriptional responses in endothelial cells [13] | |
|  |  |  |  | |  |
| Inhibition by In_4 | STAT3_Ser727p mediated inhibition of STAT3 -Tyr705 phosphorylation | Concentration of STAT3_Ser727p = Dynamic***  Ki of STAT3_Ser727p = 6.2 E-4 μM | | pSer727 of STAT3 regulates its activity by enhancing dephosphorylation of pTyr705 through TC45 [14].  Phosphorylation of STAT3 Ser727 by CDK 1 is critical for nocodazole-induced mitotic arrest [15].  Ser phosphorylation and negative regulation of Stat3 by JNK [16].  STAT3 Ser phosphorylation by ERK-dependent and independent pathways negatively modulates its Tyr phosphorylation [17] | |

*Concentration of complex A (EGFR dimerized phosphorylated and bound to JAK1 and JAK2) is dynamically generated in model when EGFR is activated by ligand and undergoes these molecular processes.

**Concentrations of In_2 (SOCS1) and In_3 (SOCS3) is dynamically generated as its transcription by factors like STAT1, STAT3 etc is modeled.

***In_4 (Ser727 phosphorylated form of STAT3) is dynamically generated in the model by phosphorylation of STAT3 at serine727 by kinases such as CDK1, JNK, ERK etc.

**Derivation of Kinetic Parameters** **explained using alignment data**

Since parameters such as Kcat /Km/Ki are not known for signaling reactions, we had to reverse engineer them, in order to align them to the reported end-point effects in the literature. These experimental results become the alignment/training data. In the reaction node explained above, the end-point validations that had to be aligned for reverse engineering two of the parameters are illustrated in the **Box 3 and Box 4** below. Additional insights into the methodology of model development can be found in our previous publications [18-26].

**Box 3**

| **Modulation** | Strength of EGF-mediated increase in pSTAT3 |
| --- | --- |
| **Parameter** | Vf_A (Vf by the EGFR2P_JAK1_JAK2p complex) |
| **Experimental results (from published data) used for reverse engineering** | A431, HN5, and 293T-EGFR cells were transfected with the Stat3 reporter construct pAPRE-luc and allowed to adhere overnight. Cells were treated with increasing concentrations of EGF (0 – 50 ng/ml) for a further 24 h, lysed and assessed for luciferase activity. Ref: [27] |
|  | A431 were treated with AG1478 (0, 0.4 and 2 μM) for 30 min in serum-free media, then stimulated with or without EGF (20 ng/ml). Ref: [27] |
| **Alignment Goal** | Vf_A to be given a value such that 20 ng/mL EGF causes ~10 fold increase in active STAT3p. |

**Box 4**

| **Modulation** | Strength of Inhibition of STAT3 phosphorylation (Tyr705) by PIAS3 |
| --- | --- |
| **Parameter** | Ki_In_1 ( Ki of PIAS3) |
| **Experimental results (from published data) used for reverse engineering** | Transfected increasing concentration of PIAS3 expression constructs or the empty pCMV5 vector transfected in A549 cells and then stimulated with EGF. After 48 h of transfection, cells were harvested, nuclear extracts were prepared and immunoblotted for pSTAT3.  Ref: [5] |
|  | PIAS3 significantly inhibits STAT3 regulated gene expression in WT STAT3 but fails to do so in cells transfected with Y705F mutant of STAT3. After 48 h of co-transfection with WT or Y705F mutant with luciferase reporter pTA-LUC vector or empty pTA-LUC vector with PIAS3 or without PIAS3, cells were triggered with EGF for 10 min. The luciferase activities of WT and mutant were compared with control (C), which is without PIAS3 transfection.  Ref: [5] |
| **Alignment Goal** | Ki_In_1 to be given a value such that 5-fold increase in PIAS3 (0.2 - 1 μg) causes ~60% decrease in Tyr705 phosphorylated STAT3 |

1. **Definition of Disease Phenotypes**

There are a few indices defined in the system to correlate to the phenotypes of cancer cell such as proliferation, viability etc. The indices are average functions of biomarkers that have been implicated in regulating these aspects of the tumor cell. The proliferation index is an average function of active CDK-cyclin complexes that define cell cycle checkpoints and are key for regulating overall tumor proliferation potential. The biomarkers include CDK4-CCND1, CDK2-CCNE, CDK2-CCNA and CDK1-CCNB1. The biomarkers have been given a certain weightage and their permutations are used for index definition so as to produce maximum correlation with experimentally reported trends for proliferation.

The viability index is a function of two sub-indices: Survival and Apoptosis. The components for each of the sub-indices have been selected based on their regulation and convergence towards these end points. The biomarkers that comprise the Survival Index include: AKT1, BCL2, MCL1, BIRC5, BIRC2, and XIAP. All these biomarkers are well reported to support tumor survival. The apoptosis index comprises BAX, CASP3, NOXA, and CASP8. Caspase 8 is the main caspase that mediates the extrinsic apoptotic pathway. BAX and CASP3 are the major apoptotic proteins that converge the extrinsic and intrinsic apoptotic pathways. NOXA regulates key survival proteins downstream by inhibiting their activity and thereby regulating cell apoptosis. The overall cell viability index is then calculated as a ratio of Survival Index/Apoptosis Index. The weightage of each biomarker is defined so as to reach a maximum correlation with well-known trends for the end points.

In order to correlate results from experiments such as MTT assay that capture information on number of viable cells and proliferation, an index called Relative Growth Index (RGI) is used – an average of the Survival and Proliferation indices.

The percentage change in these indices following a therapeutic intervention helps assess the impact of that therapy on the tumor cell. A decrease in the Proliferation/Viability Index of <20% following drug application is considered resistance, whereas a decrease of >20% indicates sensitivity to the drug.

1. **Model Validations**

The highlight of our *in silico* model is its ability to capture the emergent behavior of the cell as a whole – owing to extensive coverage of the various pathways and their cross-talks included in the simulations. Once the model version is finalized, it is validated against an extensive set of experimental results reported in published scientific literature. New validation studies are added to this regression suite with every new sub-version. The suite of validation studies includes prospective and retrospective validations and comparison of the predictions with biomarker and phenotype assays in cell lines, animals and even human studies. This is done using an engineering-based regression methodology where hundreds of studies are tested on the system. Some of our previous publications that report prospective/retrospective validation of results obtained from simulation model have been enlisted in support of validation of the *in silico* tumor model used in the study [23, 28-30].

1. ***In silico* Creation of Cell Lines and Variants**

The *in silico* model at its “baseline” is representative of a non-transformed epithelial cell. To create the simulation equivalent of a cancer cell line, it is triggered with genetic perturbations like mutation and copy number variation (CNV) data known for that cell line. To test different gene mutation-drug response associations reported in the Garnett study [31], we created cell line variants by adding or removing specific mutations from the parent cell line definition to study the impact of the perturbation. To compare the effect of drug across these different phenotypes, the change in phenotypic indices/markers between the control and disease states in each case was normalized to 100% and accordingly the effect of a drug was calculated.

1. **Simulation of Drug Effect**

To simulate the effect of a drug in the *in silico* tumor model, the targets and mechanisms of action of the drug are determined from published literature. The drug concentration is explicitly assumed to be post-ADME (Absorption, Distribution, Metabolism and Excretion). For example, PD0332991 is a selective inhibitor of cyclin-dependent kinases CDK4 and CDK6. CDK4 is known to catalyze the phosphorylation of RB1, RBL1, FOXM1 and DMTF1. Similarly CDK6 is known catalyze the phosphorylation of FOXM1. The Vf in these fluxes are inhibited by a factor (1+[I]/Ki) where [I] is drug concentration and Ki is the experimentally reported inhibition constant of the drug. IC_50_ of PD0332991 for CDK4/cyclinD1 is 0.011 μM and that for CDK6/CCND2 is 0.015 μM [32].

1. **Creation of Simulation Avatars of Patient-Derived GBM Cell Lines**

To create the simulation equivalent of a patient’s tumor cell, we used genomic data from patient-derived GBM cell lines. We triggered the model with genetic perturbations like mutation and copy number variation (CNV) data known for patient-derived cells.

We considered genes relevant for cancer – those above the threshold for gain of copy number (probe median> 0.75) were taken to be over-expressed, while those below the threshold for loss (probe median <-0.75) were taken to be knocked-down. Of all the cancer genes meeting the above criteria, those supported in the current technology version were included to create the profile definitions for patient-derived cell lines (**Supplementary Table 1**). Similar to the protocol followed for cell line creation, we created simulation models of patient lines by incorporating specific genetic perturbations on to the control network. For e.g., SK987 cell lines had overexpression of AKT1, EGFR, IL6, PI3K, etc. and knockdown of CDKN2A, CDKN2B, RUNX3, etc. (**Supplementary Table 1**). We used this data to generate *in silico* profiles of patient-derived cell lines. Further, the magnitude of gene perturbation was optimized based on the response of these cells *in vitro* to three molecularly targeted agents erlotinib, sorafenib and dasatinib. The responses of the cells to these drugs *in vitro* were used as the alignment data set.

**Alignment with Three Drug Agents**

We used 3 drugs – erlotinib, sorafenib and dasatinib, to “align” the simulation models of patient-derived GBM cell lines. Using only expression data for simulation of patient-derived cells does not provide an accurate measure of the dominance of different intracellular signaling pathways. In order to interrogate this information on the dominance of key pathways such as EGFR, RAS and PI3K signaling, we used the 3 drugs that target these pathways. This helped us align and train the simulation avatars for further analyses.

For instance, most patient-derived GBM cell lines had a gain in EGFR gene copy number. However, the effect of the EGFR inhibitor, erlotinib on cells would be a good indicator of the relative dominance of the EGFR pathway in driving the cancer cell growth in these cells. Similarly sorafenib, a RAF inhibitor could give an insight into the dominance of the RAF/MEK/ERK signaling vis-à-vis other signaling axes such as PI3K/AKT/mTOR pathway. The SRC inhibitor, dasatinib is indicative of the dominance of SRC-mediated signaling, which is increased in cancers. SRC also forms a positive feed forward loop by activating EGFR, a receptor overexpressed in gliomas.

The three alignment drugs were simulated on the *in silico* model and their responses were compared with the experimentally determined drug responses on the patient-derived GBM cells *in vitro*. The profile definitions of these cells were optimized in terms of their amplitudes of over-expression or knockdown of the specific mutations to obtain a correlation with the experimental readouts for the alignment drug agents. To illustrate this process we describe the alignment done in case of two cell lines – SK987 and SK748.

Both SK987 and SK748 demonstrated EGFR overexpression. However, erlotinib treatment showed ~85% decrease in viability in SK987 as compared to no decrease in viability in SK748 cells *in vitro*. Based on this observation, we infer that the “dominance” of EGFR signaling in SK987 should be much higher than that in SK748 cells. Further, resistance to erlotinib in SK748 cells could be due to presence of PTEN knockdown in this profile, as it has been reported earlier that PTEN loss may cause resistance to anti-EGFR therapy. So, it can be concluded that tumorigenesis in SK748 may be driven by other mediators, possibly including PTEN loss leading to increase in PI3K🡪AKT signaling. On the other hand, the decrease in cell viability with erlotinib (despite the presence of PIK3CA and AKT overexpression) in SK987 suggests that tumorigenesis in these cells may be driven by EGFR. Hence we used the alignment drugs to train the *in silico* model to get the best alignment of the simulation avatars of patient-derived GBM cell lines with experimental results available.

We created simulation avatars for patient-derived GBM cell lines with the data available on copy number variation. However, additional information on somatic mutations and epigenetic changes like methylation status of genes, if available, would enable us to create simulation avatars with higher *in silico* predictability.

Following creation and optimization of the *in silico* avatars, through blinded prospective simulation studies, we tested and compared *in silico* predictions of ten targeted drugs on the patient-derived GBM cell lines with the experimental drug sensitivity data. The protocol followed for creation and optimization of the eight GBM cell line profiles has been summarized as flow chart in **Supplementary Figure 6**). We determined the *in silico* response of increasing concentrations of the drugs on cell viability in the simulation avatars. We constructed a dose-response curve by assuming as base concentration C, the drug amount that achieved ~75% inhibition on primary target. We selected subsequent doses as: C/32, C/16, C/8, C/4, C/2, 2C, 4C. A decrease in the Viability Index with these increasing drug concentrations was used to predict the response of the patient-derived cell lines to 10 different drugs. For experimental data, a response of >80% cell survival compared to control (or <20% decrease in cell viability) following drug treatment was labeled as “resistant”, whereas <80% of cell survival compared to control (or >20% decrease in cell viability) was considered “sensitive”. The same cutoffs were used to label cells as sensitive or resistant to a drug in the *in silico* model.

**Supplementary References**

1. Chong CR, Janne PA: **The quest to overcome resistance to EGFR-targeted therapies in cancer.** *Nat Med* 2013, **19:**1389-1400.

2. Park OK, Schaefer TS, Nathans D: **In vitro activation of Stat3 by epidermal growth factor receptor kinase.** *Proc Natl Acad Sci U S A* 1996, **93:**13704-13708.

3. Ihle JN, Nosaka T, Thierfelder W, Quelle FW, Shimoda K: **Jaks and Stats in cytokine signaling.** *Stem Cells* 1997, **15 Suppl 1:**105-111; discussion 112.

4. Chung CD, Liao J, Liu B, Rao X, Jay P, Berta P, Shuai K: **Specific inhibition of Stat3 signal transduction by PIAS3.** *Science* 1997, **278:**1803-1805.

5. Dabir S, Kluge A, Dowlati A: **The association and nuclear translocation of the PIAS3-STAT3 complex is ligand and time dependent.** *Mol Cancer Res* 2009, **7:**1854-1860.

6. Kluge A, Dabir S, Vlassenbroeck I, Eisenberg R, Dowlati A: **Protein inhibitor of activated STAT3 expression in lung cancer.** *Mol Oncol* 2011, **5:**256-264.

7. Souma Y, Nishida T, Serada S, Iwahori K, Takahashi T, Fujimoto M, Ripley B, Nakajima K, Miyazaki Y, Mori M, et al: **Antiproliferative effect of SOCS-1 through the suppression of STAT3 and p38 MAPK activation in gastric cancer cells.** *Int J Cancer* 2012, **131:**1287-1296.

8. Park Y, Shon SK, Kim A, Kim KI, Yang Y, Cho DH, Lee MS, Lim JS: **SOCS1 induced by NDRG2 expression negatively regulates STAT3 activation in breast cancer cells.** *Biochem Biophys Res Commun* 2007, **363:**361-367.

9. Lee TL, Yeh J, Van Waes C, Chen Z: **Epigenetic modification of SOCS-1 differentially regulates STAT3 activation in response to interleukin-6 receptor and epidermal growth factor receptor signaling through JAK and/or MEK in head and neck squamous cell carcinomas.** *Mol Cancer Ther* 2006, **5:**8-19.

10. Schmitz J, Weissenbach M, Haan S, Heinrich PC, Schaper F: **SOCS3 exerts its inhibitory function on interleukin-6 signal transduction through the SHP2 recruitment site of gp130.** *J Biol Chem* 2000, **275:**12848-12856.

11. Gao Y, Cimica V, Reich NC: **Suppressor of cytokine signaling 3 inhibits breast tumor kinase activation of STAT3.** *J Biol Chem* 2012, **287:**20904-20912.

12. Liang P, Cheng SH, Cheng CK, Lau KM, Lin SY, Chow EY, Chan NP, Ip RK, Wong RS, Ng MH: **Platelet factor 4 induces cell apoptosis by inhibition of STAT3 via up-regulation of SOCS3 expression in multiple myeloma.** *Haematologica* 2013, **98:**288-295.

13. Bluyssen HA, Rastmanesh MM, Tilburgs C, Jie K, Wesseling S, Goumans MJ, Boer P, Joles JA, Braam B: **IFN gamma-dependent SOCS3 expression inhibits IL-6-induced STAT3 phosphorylation and differentially affects IL-6 mediated transcriptional responses in endothelial cells.** *Am J Physiol Cell Physiol* 2010, **299:**C354-362.

14. Wakahara R, Kunimoto H, Tanino K, Kojima H, Inoue A, Shintaku H, Nakajima K: **Phospho-Ser727 of STAT3 regulates STAT3 activity by enhancing dephosphorylation of phospho-Tyr705 largely through TC45.** *Genes Cells* 2012, **17:**132-145.

15. Shi X, Zhang H, Paddon H, Lee G, Cao X, Pelech S: **Phosphorylation of STAT3 serine-727 by cyclin-dependent kinase 1 is critical for nocodazole-induced mitotic arrest.** *Biochemistry* 2006, **45:**5857-5867.

16. Lim CP, Cao X: **Serine phosphorylation and negative regulation of Stat3 by JNK.** *J Biol Chem* 1999, **274:**31055-31061.

17. Chung J, Uchida E, Grammer TC, Blenis J: **STAT3 serine phosphorylation by ERK-dependent and -independent pathways negatively modulates its tyrosine phosphorylation.** *Mol Cell Biol* 1997, **17:**6508-6516.

18. Almine JF, Wise SG, Hiob M, Singh NK, Tiwari KK, Vali S, Abbasi T, Weiss AS: **Elastin sequences trigger transient proinflammatory responses by human dermal fibroblasts.** *FASEB J* 2013, **27:**3455-3465.

19. Barve A, Gupta A, Solapure SM, Kumar A, Ramachandran V, Seshadri K, Vali S, Datta S: **A kinetic platform for in silico modeling of the metabolic dynamics in Escherichia coli.** *Adv Appl Bioinform Chem* 2010, **3:**97-110.

20. Equils O, Nambiar P, Hobel CJ, Smith R, Simmons CF, Vali S: **A computer simulation of progesterone and Cox2 inhibitor treatment for preterm labor.** *PLoS One* 2010, **5:**e8502.

21. Harvey LE, Kohlgraf KG, Mehalick LA, Raina M, Recker EN, Radhakrishnan S, Prasad SA, Vidva R, Progulske-Fox A, Cavanaugh JE, et al: **Defensin DEFB103 bidirectionally regulates chemokine and cytokine responses to a pro-inflammatory stimulus.** *Sci Rep* 2013, **3:**1232.

22. Kaushik P, Gorin F, Vali S: **Dynamics of tyrosine hydroxylase mediated regulation of dopamine synthesis.** *J Comput Neurosci* 2007, **22:**147-160.

23. Rajendran P, Ong TH, Chen L, Li F, Shanmugam MK, Vali S, Abbasi T, Kapoor S, Sharma A, Kumar AP, et al: **Suppression of signal transducer and activator of transcription 3 activation by butein inhibits growth of human hepatocellular carcinoma in vivo.** *Clin Cancer Res* 2011, **17:**1425-1439.

24. Sultana Z, Paleologou KE, Al-Mansoori KM, Ardah MT, Singh N, Usmani S, Jiao H, Martin FL, Bharath MM, Vali S, El-Agnaf OM: **Dynamic modeling of alpha-synuclein aggregation in dopaminergic neuronal system indicates points of neuroprotective intervention: experimental validation with implications for Parkinson's therapy.** *Neuroscience* 2011, **199:**303-317.

25. Vali S, Mythri RB, Jagatha B, Padiadpu J, Ramanujan KS, Andersen JK, Gorin F, Bharath MM: **Integrating glutathione metabolism and mitochondrial dysfunction with implications for Parkinson's disease: a dynamic model.** *Neuroscience* 2007, **149:**917-930.

26. Vali S, Pallavi R, Kapoor S, Tatu U: **Virtual prototyping study shows increased ATPase activity of Hsp90 to be the key determinant of cancer phenotype.** *Syst Synth Biol* 2010, **4:**25-33.

27. Luwor R, Taylor LA, Wang B, Zhu H-J: **Tumor-associated EGFR over-expression specifically activates Stat3 and Smad7 resulting in desensitization of TGF-β signaling.** *Nature Precendings* 2008.

28. Cirstea D, Hideshima T, Rodig S, Santo L, Pozzi S, Vallet S, Ikeda H, Perrone G, Gorgun G, Patel K, et al: **Dual inhibition of akt/mammalian target of rapamycin pathway by nanoparticle albumin-bound-rapamycin and perifosine induces antitumor activity in multiple myeloma.** *Mol Cancer Ther* 2010, **9:**963-975.

29. Kannaiyan R, Hay HS, Rajendran P, Li F, Shanmugam MK, Vali S, Abbasi T, Kapoor S, Sharma A, Kumar AP, et al: **Celastrol inhibits proliferation and induces chemosensitization through down-regulation of NF-kappaB and STAT3 regulated gene products in multiple myeloma cells.** *Br J Pharmacol* 2011, **164:**1506-1521.

30. Tandon R, Kapoor S, Vali S, Senthil V, Nithya D, Venkataramanan R, Sharma A, Talwadkar A, Ray A, Bhatnagar PK, Dastidar SG: **Dual epidermal growth factor receptor (EGFR)/insulin-like growth factor-1 receptor (IGF-1R) inhibitor: a novel approach for overcoming resistance in anticancer treatment.** *Eur J Pharmacol* 2011, **667:**56-65.

31. Garnett MJ, Edelman EJ, Heidorn SJ, Greenman CD, Dastur A, Lau KW, Greninger P, Thompson IR, Luo X, Soares J, et al: **Systematic identification of genomic markers of drug sensitivity in cancer cells.** *Nature* 2012, **483:**570-575.

32. Fry DW, Harvey PJ, Keller PR, Elliott WL, Meade M, Trachet E, Albassam M, Zheng X, Leopold WR, Pryer NK, Toogood PL: **Specific inhibition of cyclin-dependent kinase 4/6 by PD 0332991 and associated antitumor activity in human tumor xenografts.** *Mol Cancer Ther* 2004, **3:**1427-1438.

**Supplementary Figure Legends**

**Supplementary Figure 1**: Schematic representing the important signaling cascades activated by binding of EGF to its receptor. Circles: Species (proteins, metabolites etc. in cell); Square box: Reaction nodes where reaction equation and parameters are defined; Grey Arrows: Flux arrows that show conversion of one form of a species to another; Green arrows: activation; Red Arrows: inhibition.

**Supplementary Figure 2**: Snapshot demonstrating basic signaling of the EGFR pathway, as represented in the *in silico* model.

**Supplementary Table Legends**

Supplementary Table 1: **Profiling information for creation of patient-derived GBM lines *in silico*.** Cancer genes above the threshold for gain of expression (probe median > 0.75) were taken to be overexpressed, while those below the threshold for loss (probe median <-0.75) were taken to be knocked down. Of all the cancer genes meeting the above criteria, those supported in the current technology version were included in the profile definitions. Genes highlighted in the grey background are overexpressed in the given profile definitions of patient-derived GBM cell lines; all other genes enlisted are knocked down.

Supplementary Table 2: **Cell lines in the *in silico* model library**

Supplementary Table 3: **Drugs supported in the *in silico* tumor model**

Supplementary Table 4: **Genes represented in the *in silico* model that have been screened for mutations in the Garnett study**

Supplementary Table 5: **Retrospective validation of the *in silico* tumor model by testing gene mutation-drug response associations discussed in the Garnett study.** This table depicts agreement/difference between *in silico* simulation analyses and data reported in the Garnett study. For correlations extracted from Supplementary Data 4 of the Garnett study, threshold for inclusion of gene mutation-drug response was p-value <1E-4. (OE - Overexpression)

Supplementary Table 6: **Gene mutation-drug response correlations in the Garnett study currently not supported by our technology**.

Supplementary Table 7: **Prospective Validation – Comparing *in silico* predicted drug responses in simulations of 8 patient-derived GBM cell lines with experimentally determined response (*in vitro*)**. For experimental data, a response of >80% cell survival compared to control (<20% decrease in cell viability) in response to a drug was considered as resistant to drug – R; <80% cell survival compared to control (>20% decrease in cell viability) was considered as sensitive to drug – S. For *in silico* predictions, same cutoffs were used to label cells as resistant (R) or sensitive (S) to a drug.

**Supplementary Tables**

| **SK102** | **SK987** | **SK262** | **GBM4** | **GBM8** | **SK429** | **SK748** | **SK1035** | |
| --- | --- | --- | --- | --- | --- | --- | --- | --- |
| ABI1 | BCL6 | ABI1 | ABI1 | BCL2 | AKT1 | CDKN2A | ABI1 | ABCB1 |
| ANXA2 | CDKN2A | CASP7 | BAG4 | CDKN2A | BRCA2 | CDKN2B | ANXA2 | BAG4 |
| BCL6 | CDKN2B | CDKN2A | CASP7 | CDKN2B | CDKN2A | FAS | BRCA1 | CAV1 |
| CDKN2A | RUNX3 | CDKN2B | CHUK | DYRK2 | CDKN2B | IFNA | BRCA2 | CTSB |
| CDKN2B | VEGFC | CHUK | CTSB | IFNA | FOS | IKBKE | CDKN2A | EGFR |
| CHUK | AKT1 | CXCL12 | CXCL12 | NFKBIA | FOXO1 | PTEN | CDKN2B | ETV1 |
| CSK | BCL2 | DKK1 | DKK1 | SERPINB2 | HIF1A | EGFR | CHUK | EZH2 |
| CXCL12 | CDH2 | EZR | FAS | ABCB1 | HSP90AA1 |  | CXCL12 | FGFR |
| DKK1 | EGFR | FAS | FGFR | BTK | NFKBIA |  | DKK1 | GLI3 |
| FAS | ETV1 | FGFR2 | FGFR2 | CAV1 | TCL1A |  | FAS | HAS2 |
| FES | IFNA | FRAT1 | FRAT1 | CD40LG | TNFSF11 |  | FGFR2 | IKBKB |
| FGFR2 | IL6 | GATA3 | GATA3 | EGFR | TRAF3 |  | FLT1 | IL6 |
| FRAT1 | LAMA3 | IFNA | HAS2 | ETV1 | BTK_P |  | FOXO1 | LCN2 |
| GATA3 | NFKBIA | ITGB1 | IKBKB | EZH2 | CD40LG |  | FRAT1 | LOXL2 |
| IFNA | PIK3CA | JAK1 | ITGB1 | GLI3 | EZH2 |  | GATA3 | LYN |
| IGF1R | SERPINB2 | JAK2 | LYN | GPC3 | GPC3 |  | IFNA | MET |
| ITGB1 | SMAD2 | JUN | MAP3K8 | IKBKG | IKBKG |  | ITGB1 | MYC |
| MAP3K8 | SMAD4 | MAP3K8 | MAPK8 | IL6 | NOS3 |  | JAK2 | NOS3 |
| MAPK8 | YES1 | MAPK8 | MKI67 | MDM2 | SHH |  | MAP3K8 | NSMAF |
| MKI67 |  | MKI67 | NSMAF | MET |  |  | MAPK8 | PIK3CG |
| PC7 |  | NF1 | PTEN | MYCN |  |  | MKI67 | PTK2 |
| PTEN |  | PTEN | PTK2 | NOS3 |  |  | NOTCH1 | PTK2B |
| SUFU |  | SOD2 | PTK2B | PDGFRA |  |  | PC7 | RRM2B |
| ABCB1 |  | SUFU | SUFU | PIK3CG |  |  | PTEN | SHH |
| CAV1 |  | BTK | TNFRSF10A | SERPINE1 |  |  | STAT3 | SMO |
| EGFR |  | CD40LG | TNFRSF10B | SHH |  |  | SUFU | TBXAS1 |
| ETV1 |  | CEBPA | BTK |  |  |  | TRAF2 | TNFRSF10A |
| GLI3 |  | ETV1 | CD40LG |  |  |  |  | TNFRSF10B |
| HTERT |  | GPC3 | GPC3 |  |  |  |  |  |
| IL6 |  | PIK3CG | IKBKG |  |  |  |  |  |
| MET |  | SMO | JAK2 |  |  |  |  |  |
| PIK3CA |  |  | MYC |  |  |  |  |  |
| PIK3CG |  |  |  |  |  |  |  |  |

**Supplementary Table 1: Profiling information for creation of patient-derived GBM lines *in silico***

**Supplementary Table 2: Cell lines in the *in silico* model library**

| **Sl. No.** | **Cell Line** | **Cosmic ID** | **Tissue** | **Cancer-type** |
| --- | --- | --- | --- | --- |
| 1 | MDA-MB-231 | 905960 | breast | Breast |
| 2 | A172 | 687563 | CNS | Glioma |
| 3 | U-87-MG | 687590 | CNS | Glioma |
| 4 | U251 | 905983 | CNS | Glioma |
| 5 | HCT-116 | 905936 | GI tract | Large Intestine |
| 6 | HT-29 | 905939 | GI tract | Large Intestine |
| 7 | COLO-205 | 905961 | GI tract | Large Intestine |
| 8 | SW620 | 905962 | GI tract | Large Intestine |
| 9 | KM12 | 905989 | GI tract | Large Intestine |
| 10 | LoVo | 907790 | GI tract | Large Intestine |
| 11 | SW48 | 909751 | GI tract | Large Intestine |
| 12 | COLO-320-HSR | 910569 | GI tract | Large Intestine |
| 13 | SK-HEP-1 | 909719 | other | Liver |
| 14 | SNU-387 | 909736 | other | Liver |
| 15 | NCI-H1437 | 687794 | lung | Lung: NSCLC: Adenocarcinoma |
| 16 | NCI-H1650 | 687800 | lung | Lung: NSCLC: Adenocarcinoma |
| 17 | Calu-6 | 724859 | lung | Lung: NSCLC: Adenocarcinoma |
| 18 | NCI-H292 | 753604 | lung | Lung: NSCLC: Adenocarcinoma |
| 19 | NCI-H441 | 908460 | lung | Lung: NSCLC: Adenocarcinoma |
| 20 | NCI-H358 | 908465 | lung | Lung: NSCLC: Adenocarcinoma |
| 21 | NCI-H1703 | 908474 | lung | Lung: NSCLC: Adenocarcinoma |
| 22 | NCI-H1975 | 924244 | lung | Lung: NSCLC: Adenocarcinoma |
| 23 | NCI-H1299 | 724831 | lung | Lung: NSCLC: large cell |
| 24 | NCI-H460 | 905943 | lung | Lung: NSCLC: large cell |
| 25 | NCI-H1155 | 908467 | lung | Lung: NSCLC: large cell |
| 26 | NCI-H23 | 905942 | lung | Lung: NSCLC: NOS |
| 27 | NCI-H522 | 905944 | lung | Lung: NSCLC: NOS |
| 28 | A549 | 905949 | lung | Lung: NSCLC: NOS |
| 29 | SK-MM-2 | 753612 | blood | Myeloma |
| 30 | U-266 | 753615 | blood | Myeloma |
| 31 | RPMI-8226 | 905964 | blood | Myeloma |
| 32 | OPM-2 | 909249 | blood | Myeloma |
| 33 | KYSE-450 | 907320 | upper aero-digestive | Esophagus |
| 34 | IGROV-1 | 905968 | ovary | Ovary |
| 35 | MIA-PaCa-2 | 724870 | pancreas | Pancreas |
| 36 | BxPC-3 | 906693 | pancreas | Pancreas |
| 37 | DU-145 | 905935 | other | Pancreas |
| 38 | AGS | 906790 | GI tract | Stomach |
| 39 | HGC-27 | 907055 | GI tract | Stomach |
| 40 | MKN1 | 908138 | GI tract | Stomach |
| 41 | MKN28 | 908139 | GI tract | Stomach |
| 42 | NCI-SNU-1 | 908444 | GI tract | Stomach |
| 43 | NUGC-3 | 908455 | GI tract | Stomach |
| 44 | MKN45 | 925340 | GI tract | Stomach |
| 45 | BHY | 753535 | upper aero-digestive | Upper Aero-digestive tract |

**Supplementary Table 3: Drugs supported in the *in silico* tumor model**

| **Sl. No.** | **Drug name** | **Synonyms** | **Brand name** | **Drug Target(s)** |
| --- | --- | --- | --- | --- |
| 1 | Erlotinib |  | Tarceva | EGFR |
| 2 | Rapamycin | AY-22989, Sirolimus, WY-090217 | Rapamune | MTOR |
| 3 | Sunitinib | Sutent | Sutent | PDGFRA, PDGFRB, VEGFR(KDR), KIT, FLT3 |
| 4 | PHA-665752 |  |  | MET |
| 5 | MG-132 | zLLL |  | Proteasome |
| 6 | Cyclopamine | 11-deoxojervine |  | SMO |
| 7 | AZ628 |  |  | BRAF |
| 8 | Sorafenib | BAY-43-9006, Nexavar | Nexavar | PDGFRA, PDGFRB, VEGFR(KDR), KIT, FLT3 |
| 9 | VX-680 | MK-045, MK-0457, VX-68 | MK-0457 | Aurora A/B/C, FLT3, ABL1, JAK2, |
| 10 | Imatinib | Gleevec, STI-571 | Gleevec | ABL, KIT, PDGFR |
| 11 | PF-02341066 | Crizotinib, KIN001-023 |  | MET, ALK |
| 12 | Z-LLNle-CHO | Z-L-Norleucine-CHO | na | g-secretase |
| 13 | Dasatinib | KIN001-005 | Sprycel | ABL, SRC, KIT, PDGFR |
| 14 | CGP-60474 | KIN001-019 |  | CDK1/2/5/7/9 |
| 15 | CGP-082996 | CINK4, KIN001-021 |  | CDK4 |
| 16 | BMS-536924 | KIN001-126 | BMS-536924 | IGF1R |
| 17 | GW843682X | KIN001-134 | GW843682X (AN-13) | PLK1 |
| 18 | MS-275 | MS275 |  | HDAC |
| 19 | Parthenolide |  |  | NFKB1 |
| 20 | Bortezomib | LDP-341, PS-341 |  | Proteasome |
| 21 | Roscovitine | Seliciclib |  | CDKs |
| 22 | Lapatinib |  |  | EGFR, ERBB2 |
| 23 | A-769662 |  |  | AMPK agonist |
| 24 | AZD6482 | AZD6482 (KIN001-193) |  | PI3Kb (P3C2B) |
| 25 | PF-562271 | (KIN001-205) |  | FAK |
| 26 | DMOG | Dimethyloxalylglcine |  | Prolyl-4-Hydroxylase |
| 27 | Embelin |  |  | XIAP |
| 28 | PAC-1 | PAC-1 |  | CASP3 activator |
| 29 | Thapsigargin |  |  | ATPase, Ca++ transporting, cardiac muscle, slow twitch 2 |
| 30 | Obatoclax Mesylate |  | GX15-070 | BCL-2, BCL-XL, MCL-1 |
| 31 | BMS-754807 |  |  | IGF1R |
| 32 | OSI-906 | OSI-906 |  | IFG1R |
| 33 | Bexarotene | LG-100069, LGD-1069 | Targretin | Retinioic acid X family agonist |
| 34 | Bleomycin |  |  | DNA damage |
| 35 | AUY922 | VER-52296, NVP-AUY922 |  | HSP90 |
| 36 | Pazopanib | GW786034 | Votrient | VEGFR, PDGFRA, PDGFRB, KIT |
| 37 | Metformin | 1, 1-Dimethylbiguanide hydrochloride | Metformin | AMPK agonist |
| 38 | AICAR | N1-(b-D-Ribofuranosyl)-5-aminoimidazole-4-carboxamide | AICAR | AMPK agonist |
| 39 | Cisplatin | cis-Diammineplatinum(II) dichloride | Cisplatin | DNA crosslinker |
| 40 | Gefitinib | ZD-1839 | Iressa | EGFR |
| 41 | ABT-263 |  |  | BCL2, BCL-XL, BCL-W |
| 42 | Vorinostat | SAHA | Vorinostat | HDAC inhibitor Class I, IIa, IIb, IV |
| 43 | Nilotinib |  |  | ABL |
| 44 | RDEA119 | RDEA119, BAY869766 |  | MEK1/2 |
| 45 | CI-1040 | PD-18435, PD-184352 |  | MEK1/2 |
| 46 | Temsirolimus | CCI-779 | Torisel | MTOR |
| 47 | ABT-888 | ABT-888 |  | PARP1/2 |
| 48 | Bosutinib | SKI-606 | Bosutinib | SRC, ABL, TEC |
| 49 | Lenalidomide | CC-5013 |  | TNF alpha |
| 50 | Axitinib | AG-013736 | Axitinib | PDGFR, KIT, VEGFR |
| 51 | AZD7762 | AZD 7762 |  | CHK1/2 |
| 52 | 17-AAG | 17-AAG | Telatinib | HSP90 |
| 53 | VX-702 |  |  | p38 |
| 54 | AMG-706 | AMG-706 | Motesanib | VEGFR, RET, c-KIT, PDGFR |
| 55 | KU-55933 |  |  | ATM |
| 56 | BIBW2992 | Tovok |  | EGFR, ERBB2 |
| 57 | GDC-0449 | RG3616 | Vismodegib | SMO |
| 58 | PLX4720 |  |  | BRAF |
| 59 | Nutlin-3a | Nutlin-3a (-) enantiomer |  | MDM2 |
| 60 | PD-173074 | PD-173074 |  | FGFR1/3 |
| 61 | ZM-447439 | ZM447439 |  | AURKB |
| 62 | RO-3306 |  |  | CDK1 |
| 63 | MK-2206 |  |  | AKT1/2 |
| 64 | PD-0332991 | PD-0332991 |  | CDK4/6 |
| 65 | NVP-BEZ235 | BEZ235 |  | PI3K (Class 1) and mTORC1/2 |
| 66 | GDC0941 |  |  | PI3K (class 1) |
| 67 | AZD8055 | AZD8055 |  | mTORC1/2 |
| 68 | PD-0325901 | PD-0325901 |  | MEK1/2 |
| 69 | SB590885 |  |  | BRAF |
| 70 | AZD6244 | ARRY-142886 | Selumetinib | MEK1/2 |

**Supplementary Table 4: Genes represented in the *in silico* model that have been screened for mutations in the Garnett study**

| **Sl. No.** | **Genes supported** |
| --- | --- |
| 1 | APC |
| 2 | BRAF |
| 3 | BRCA1 |
| 4 | BRCA2 |
| 5 | CCND1 |
| 6 | CCND2 |
| 7 | CCND3 |
| 8 | CDH1 |
| 9 | CDK4 |
| 10 | CDKN2A |
| 11 | CDKN2C |
| 12 | CDKN2a(p14) |
| 13 | CTNNB1 |
| 14 | EGFR |
| 15 | EP300 |
| 16 | ERBB2 |
| 17 | EZH2 |
| 18 | FBXW7 |
| 19 | FGFR2 |
| 20 | FGFR3 |
| 21 | GNAS |
| 22 | IDH1 |
| 23 | JAK2 |
| 24 | KDR |
| 25 | KRAS |
| 26 | MAP2K4 |
| 27 | MDM2 |
| 28 | MET |
| 29 | MLH1 |
| 30 | MYC |
| 31 | MYCN |
| 32 | NF1 |
| 33 | NF2 |
| 34 | NOTCH1 |
| 35 | PDGFRA |
| 36 | PIK3CA |
| 37 | PIK3R1 |
| 38 | PTCH1 |
| 39 | PTEN |
| 40 | RB1 |
| 41 | SMAD4 |
| 42 | SMARCA4 |
| 43 | SMO |
| 44 | SOCS1 |
| 45 | STK11 |
| 46 | SUFU |
| 47 | TP53 |
| 48 | TSC1 |
| 49 | TSC2 |
| 50 | VHL |
| 51 | BCR_ABL |

**Supplementary Table 5: Retrospective validation of the *in silico* tumor model by testing gene mutation-drug response associations discussed in the Garnett study**

|  | **DRUG** | **TARGET** | **GENE MUTATION** | **SENSITIVITY/RESISTANCE** | **SIMULATION RESULT** |
| --- | --- | --- | --- | --- | --- |
| 1 | PLX4720 | BRAF | BRAF mutation | Sensitivity | Corroborating |
| 2 | AZD6244 | MEK | BRAF mutation | Sensitivity | Corroborating |
| 3 | Lapatinib | EGFR, ERBB2 | ERBB2 OE | Sensitivity | Corroborating |
| 4 | Lapatinib | EGFR, ERBB2 | CCND1 OE | Sensitivity | Corroborating |
| 5 | Lapatinib | EGFR, ERBB2 | MET OE | Sensitivity | Corroborating |
| 6 | Lapatinib | EGFR, ERBB2 | CDH1 mutation/loss | Sensitivity | Corroborating |
| 7 | Lapatinib | EGFR, ERBB2 | SMAD4 mutation/loss | Sensitivity | Not Corroborating |
| 8 | BIBW2992 | EGFR, ERBB2 | BRAF mutation | Resistance | Corroborating |
| 9 | Erlotinib | EGFR | CDKN2A mutation | Sensitivity | Corroborating |
| 10 | Erlotinib | EGFR | SMAD4 mutation | Sensitivity | Not Corroborating |
| 11 | PD173074 | FGFR2 | FGFR2 mutation | Sensitivity | Corroborating |
| 12 | AMG-706 | VEGFR, RET, c-KIT, PDGFR | VEGFR2 OE | Sensitivity | Corroborating |
| 13 | Dasatinib | ABL,SRC,KIT, PDGFR | CDKN2A mutation | Sensitivity | Corroborating |
| 14 | AZD6482 | PI3K | BRAF mutation | Resistance | Corroborating |
| 15 | PD0332991 | CDK4/6 | RB1 mutation | Resistance | Corroborating |
| 16 | PD0332991 | CDK4/6 | CDKN2A mutation | Sensitivity | Corroborating |
| 17 | Obatoclax | BCL2, BCL-xL, Mcl1 | BRAF mutation | Sensitivity | Corroborating |
| 18 | ABT-263 | BCL2, BCL-xL | NOTCH1 mutation | Sensitivity | Corroborating |
| 19 | ABT-263 | BCL2, BCL-xL | MCL1 OE | Resistance | Corroborating |
| 20 | Nutlin 3a | MDM2 | TP53 mutation | Resistance | Corroborating |
| 21 | Nutlin 3a | MDM2 | BRAF mutation | Sensitivity | Corroborating |
| 22 | Nutlin 3a | MDM2 | MYC OE | Sensitivity | Corroborating |
| 23 | 17-AAG | HSP90 | STK11 mutation | Sensitivity | Corroborating |
| 24 | Bortezomib | Proteasome | CDKN2A mutation | Sensitivity | Corroborating |
| 25 | Temsirolimus | mTORC1 | APC mutation | Resistance | Not Corroborating |

**Supplementary Table 6: Gene mutation-drug response correlations in the Garnett study currently not supported by our technology**

|  | **DRUG** | **TARGET** | **GENE MUTATION** | **SENSITIVITY/ RESISTANCE** | **SIMULATION DATA** |
| --- | --- | --- | --- | --- | --- |
| 1 | Nilotinib | ABL | BCR-ABL | Sensitivity | Not tested |
| 2 | Lapatinib | EGFR, ERBB2 | MMP28 OE | Sensitivity | Not tested |
| 3 | Dasatinib | ABL, SRC, KIT, PDGFR | BCR-ABL mutation | Sensitivity | Not tested |
| 4 | 17-AAG | HSP90 | NQO1 mutation | Sensitivity | Not tested |
| 5 | AZD2281 | PARP | EWS-FLI1 | Sensitivity | Not tested |
| 6 | GW441756 | NTRK1 | FLT3 | Sensitivity | Not tested |
| 7 | MS-275 | HDAC | FBXW7 loss | Sensitivity | Not tested |
| 8 | 681540 | WEE1/CHK1 | TET2 loss | Sensitivity | Not tested |
| 9 | GSK650394 | SGK3 | LAG3 OE | Sensitivity | Not tested |
| 10 | AICAR | AMPK agonist | ADK OE | Sensitivity | Not tested |
| 11 | AZD8055 | mTORC1/2 | TET2 mutant | Sensitivity | Not tested |

**Supplementary Table 7: Prospective Validation – Comparing *in silico* predicted drug responses in simulations of 8 patient-derived GBM cell lines with experimentally determined response (*in vitro*)**

a.

|  | SK102 | | | | SK987 | | | |
| --- | --- | --- | --- | --- | --- | --- | --- | --- |
|  | % Control | Exp data | Predictive data | Alignment | % Control | Exp data | Predictive data | Alignment |
| **TRAINING SET:** | | | | | | | | |
| Erlotinib | 35.83 | S | S | Match | 15.09 | S | S | Match |
| Sorafenib | 78.14 | S | S | Match | 103.21 | R | S | Mismatch |
| Dasatinib | 109.43 | R | S | Mismatch | 44.15 | S | S | Match |
| **TEST SET:** | | | | | | | | |
| Lapatinib | 51.44 | S | S | Match | 19.03 | S | S | Match |
| Nilotinib | 93.27 | R | R | Match | 105.98 | R | R | Match |
| Imatinib | 101.78 | R | R | Match | 89.83 | R | R | Match |
| Sunitinib | 98.08 | R | S | Mismatch | 110.63 | R | S | Mismatch |
| Pitavastatin | 75.01 | S | S | Match | 106.82 | R | S | Mismatch |
| Vorinostat | 47.88 | S | S | Match | 18.93 | S | S | Match |
| Bortezomib | 44.49 | S | S | Match | 7.58 | S | S | Match |
| Celecoxib | 86.60 | R | S | Mismatch | 131.12 | R | S | Mismatch |
| Everolimus | 69.54 | S | S | Match | 3.00 | S | S | Match |
| Rapamycin | 101.59 | R | R | Match | 154.66 | R | R | Match |

b.

|  | SK262 | | | | GBM4 | | | |
| --- | --- | --- | --- | --- | --- | --- | --- | --- |
|  | % Control | Exp data | Pre data | Alignment | % Control | Exp data | Pre data | Alignment |
| **TRAINING SET:** | | | | | | | | |
| Erlotinib | 60.07 | S | S | Match | 29.64 | S | S | Match |
| Sorafenib | 59.82 | S | S | Match | 76.61 | S | R | Mismatch |
| Dasatinib | 60.05 | S | S | Match | 109.13 | R | R | Match |
| **TEST SET:** | | | | | | | | |
| Lapatinib | 10.34 | S | S | Match | 25.01 | S | R | Mismatch |
| Nilotinib | 64.61 | S | R | Mismatch | 99.55 | R | R | Match |
| Imatinib | 100.12 | R | R | Match | 142.51 | R | R | Match |
| Sunitinib | 60.03 | S | R | Mismatch | 95.62 | R | R | Match |
| Pitavastatin | 18.09 | S | S | Match | 85.13 | R | S | Mismatch |
| Vorinostat | 25.37 | S | S | Match | 25.86 | S | S | Match |
| Bortezomib | 7.52 | S | S | Match | 23.85 | S | S | Match |
| Celecoxib | 93.30 | R | R | Match | 99.20 | R | R | Match |
| Everolimus | 48.30 | S | S | Match | 67.57 | S | S | Match |
| Rapamycin | 37.52 | S | R | Mismatch | 158.08 | R | R | Match |

c.

|  | GBM8 | | | | SK429 | | | |
| --- | --- | --- | --- | --- | --- | --- | --- | --- |
|  | % Control | Exp data | Pre data | Alignment | % Control | Exp data | Pre data | Alignment |
| **TRAINING SET:** | | | | | | | | |
| Erlotinib HCl | 19.12 | S | S | Match | 84.34 | R | R | Match |
| Sorafenib | 6.35 | S | S | Match | 91.47 | R | R | Match |
| Dasatinib | 12.75 | S | S | Match | 157.36 | R | R | Match |
| **TEST SET:** | | | | | | | | |
| Lapatinib | 7.39 | S | S | Match | 87.44 | R | R | Match |
| Nilotinib | 14.05 | S | S | Match | 115.09 | R | R | Match |
| Imatinib | 26.65 | S | S | Match | 109.82 | R | R | Match |
| Sunitinib | 7.55 | S | S | Match | 112.76 | R | R | Match |
| Pitavastatin | 6.49 | S | S | Match | 77.39 | S | R | Mismatch |
| Vorinostat | 6.34 | S | S | Match | 53.11 | S | S | Match |
| Bortezomib | 6.41 | S | S | Match | 58.02 | S | S | Match |
| Celecoxib | 80.92 | R | S | Mismatch | 88.32 | R | R | Match |
| Everolimus | 7.66 | S | S | Match | 138.19 | R | S | Mismatch |
| Rapamycin | 7.10 | S | R | Mismatch | 71.49 | S | R | Mismatch |

d.

|  | SK748 | | | | SK1035 | | | |
| --- | --- | --- | --- | --- | --- | --- | --- | --- |
|  | % Control | Exp data | Pre data | Alignment | % Control | Exp data | Pre data | Alignment |
| **TRAINING SET:** | | | | | | | | |
| Erlotinib HCl | 109.03 | R | R | Match | 96.61 | R | R | Match |
| Sorafenib | 84.46 | R | R | Match | 94.13 | R | R | Match |
| Dasatinib | 81.29 | R | R | Match | 89.93 | R | R | Match |
| **TEST SET:** | | | | | | | | |
| Lapatinib | 87.51 | R | R | Match | 99.64 | R | R | Match |
| Nilotinib | 85.80 | R | R | Match | 78.43 | S | R | Mismatch |
| Imatinib | 120.16 | R | R | Match | 122.13 | R | R | Match |
| Sunitinib | 106.51 | R | R | Match | 117.12 | R | R | Match |
| Pitavastatin | 100.00 | R | R | Match | 12.34 | S | R | Mismatch |
| Vorinostat | 89.55 | R | S | Mismatch | 66.62 | S | S | Match |
| Bortezomib | 18.31 | S | S | Match | 23.03 | S | S | Match |
| Celecoxib | 112.71 | R | R | Match | 97.77 | R | R | Match |
| Everolimus | 69.50 | S | S | Match | 81.65 | R | S | Mismatch |
| Rapamycin | 86.97 | R | R | Match | 90.97 | R | R | Match |
